# Hotspot Mutations in SARS-CoV-2

**DOI:** 10.3389/fgene.2021.753440

**Published:** 2021-11-29

**Authors:** Indrajit Saha, Nimisha Ghosh, Nikhil Sharma, Suman Nandi

**Affiliations:** ^1^ Department of Computer Science and Engineering, National Institute of Technical Teachers’ Training and Research, Kolkata, India; ^2^ Department of Computer Science and Information Technology, Institute of Technical Education and Research, Siksha ‘O’ Anusandhan (Deemed to be University), Bhubaneswar, India; ^3^ Department of Electronics and Communication Engineering, Jaypee Institute of Information Technology, Noida, India

**Keywords:** COVID-19, deletions, entropy, hotspot mutations, SARS-CoV-2 genomes, substitution

## Abstract

Since its emergence in Wuhan, China, severe acute respiratory syndrome coronavirus-2 (SARS-CoV-2) has spread very rapidly around the world, resulting in a global pandemic. Though the vaccination process has started, the number of COVID-affected patients is still quite large. Hence, an analysis of hotspot mutations of the different evolving virus strains needs to be carried out. In this regard, multiple sequence alignment of 71,038 SARS-CoV-2 genomes of 98 countries over the period from January 2020 to June 2021 is performed using MAFFT followed by phylogenetic analysis in order to visualize the virus evolution. These steps resulted in the identification of hotspot mutations as deletions and substitutions in the coding regions based on entropy greater than or equal to 0.3, leading to a total of 45 unique hotspot mutations. Moreover, 10,286 Indian sequences are considered from 71,038 global SARS-CoV-2 sequences as a demonstrative example that gives 52 unique hotspot mutations. Furthermore, the evolution of the hotspot mutations along with the mutations in variants of concern is visualized, and their characteristics are discussed as well. Also, for all the non-synonymous substitutions (missense mutations), the functional consequences of amino acid changes in the respective protein structures are calculated using PolyPhen-2 and I-Mutant 2.0. In addition to this, SSIPe is used to report the binding affinity between the receptor-binding domain of Spike protein and human ACE2 protein by considering L452R, T478K, E484Q, and N501Y hotspot mutations in that region.

## 1 Introduction

COVID-19 caused by severe acute respiratory syndrome coronavirus-2 (SARS-CoV-2) was first identified in late December 2019 and has a high transmission rate (Zhu et al., 2020). The WHO declared this outbreak as a pandemic on March 11, 2020 (Cucinotta and Vanelli, 2020). Like other coronaviruses, SARS-CoV-2 is also an enveloped single-stranded RNA virus containing nearly 30 K nucleotide sequences (Alexandersen et al., 2020). SARS-CoV-2 encompasses 11 codding regions, which include ORF1ab, Spike (S), ORF3a, Envelope (E), Membrane (M), ORF6, ORF7a, ORF7b, ORF8, Nucleocapsid (N), and ORF10.

Though the vaccination process has started, the virus is evolving and spreading all across the world, causing fresh waves every few months. Since the virus is mutating frequently, it creates new variant of the original virus. Among several variants, B.1.1.7 (Alpha), B.1.351 (Beta), P.1 (Gamma), and B.1.617.2 (Delta) are declared as variants of concern (Singh et al., 2021). In this regard, the variant B.1.1.7 was first identified in the United Kingdom, which contains E484K, N501Y, D614G, and P681H mutations in Spike glycoprotein (Tang et al., 2020). In December 2020, the variant B.1.351 was first detected in South Africa, with mutations such as K417N, E484K, N501Y, D614G, and A701V (Tang et al., 2021). The Brazilian variant P.1 also has almost the same mutations as the B.1.351 variant, but instead of A701V, the P.1 variant has H555Y mutation (Faria et al., 2021). On the other hand, the variant B.1.617.2 was first identified in India with L452R, T478K, D614G, and P681R mutations in Spike glycoprotein (Bernal et al., 2021).

To understand the new variants of SARS-CoV-2, Tiwari and Mishra (2021) have performed phylogenetic analysis of 591 SARS-CoV-2 genomes where they have found 43 synonymous and 57 non-synonymous mutations in 12 protein regions. They found the most prevalent mutations in the Spike protein, followed by NSP2, NSP3, and ORF9. They have also highlighted several distinct SARS-CoV-2 features as compared with other human-infecting viruses. Yuan et al. (2020) have analyzed 11,183 global sequences where they have identified 119 single-nucleotide polymorphisms (SNPs) with 74 non-synonymous and 43 synonymous mutations. The mutational profiling shows that the highest mutation has occurred in Nucleocapsid, followed by NSP2, NSP3, and Spike. From China, India, the United States, and Europe, 570 SARS-CoV-2 genomes are analyzed by Weber et al. (2020), where they have identified 10 individual mutations where most of the mutations altered the amino acids in the replication-relevant proteins. Sarkar et al. (2021) have performed a genome-wide analysis of 837 Indian SARS-CoV-2 genomes, where 33 unique mutations were observed, among which 18 mutations were identified in India in five protein regions (six in Spike, five in NSP3, four in RdRp, two in NSP2, and one in Nucleocapsid). The isolated Indian sequences were classified into 22 groups based on their coexisting mutations. This study highlights several mutations identified in various protein regions, which also help to identify the evolution of virus genome across various geographic locations of India. Saha et al. (2020) have performed phylogenetic analysis of 566 Indian SARS-CoV-2 genomes to identify several mutations. As a result, 933 substitutions, 2,449 deletions, and two insertions have been identified from the aligned sequences. In another study, Saha et al. (2021) have performed genomic analysis of 10,664 SARS-CoV-2 genomes, resulting in 7,209 substitutions, 11,700 deletions, 119 insertions, and 53 SNPs.

Motivated by the aforementioned analysis, in this work, we have performed multiple sequence alignment (MSA) of 71,038 SARS-CoV-2 genomes using MAFFT (Katoh et al., 2002) followed by their phylogenetic analysis using Nextstrain (Hadfield et al., 2018) to visualize the virus evolution. This led to the identification of hotspot mutations as deletions and substitutions in the coding regions based on entropy greater than or equal to 0.3. Furthermore, as a demonstrative example, 10,286 Indian sequences are considered from 71,038 global SARS-CoV-2 sequences. For all the non-synonymous substitutions (missense mutations), the functional consequences of amino acid changes in the respective protein structures are calculated using PolyPhen-2 and I-Mutant 2.0. Finally, SSIPe is used to report the binding affinity between the receptor-binding domain (RBD) of Spike protein and human ACE2 protein by considering the hotspot mutations in that region.

## 2 Methods

In this section, the dataset collection for the SARS-CoV-2 genomes is discussed along with the proposed pipeline.

### 2.1 Data Preparation

For MSA and phylogenetic analysis, 71,038 global SARS-CoV-2 genomes are collected from Global Initiative on Sharing All Influenza Data (GISAID)^1^, and the Reference Genome (NC 045512.2)^2^ is collected from the National Center for Biotechnology Information (NCBI). The SARS-CoV-2 sequences are mostly distributed from January 2020 to June 2021 globally. Moreover, to map the protein sequences and changes in the amino acid, Protein Data Bank (PDB) is collected from Zhang Lab^3^ (Zhang et al., 2020; Wu et al., 2021), and it is then used to show the structural changes. All these analyses are performed on the High Performance Computing facility of NITTTR, Kolkata; and for checking the amino acid changes, MATLAB R2019b is used.

### 2.2 Pipeline of the Work

**FIGURE 1:**
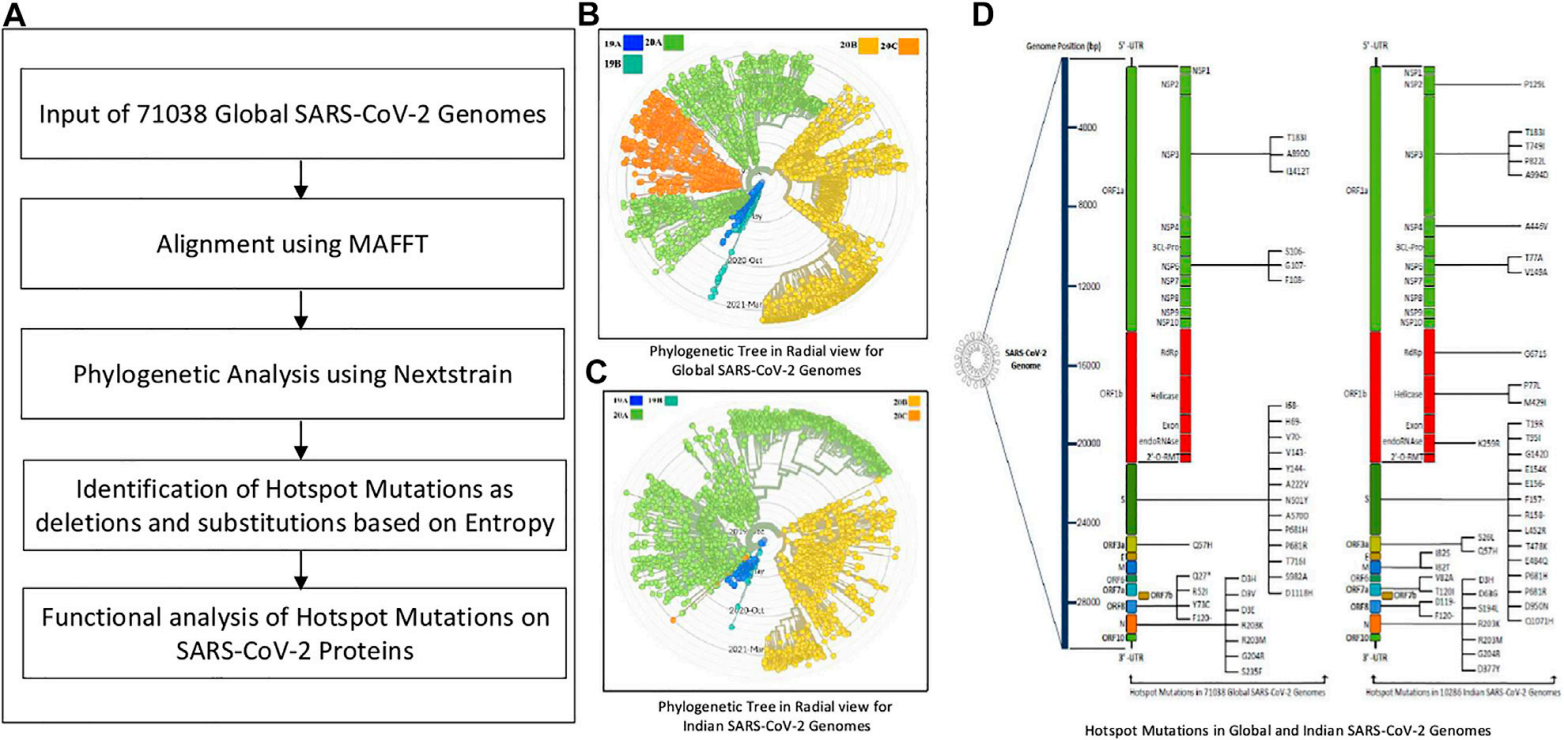
Pipeline of the workflow.

The pipeline of this work is provided in Figure 1A. Initially, MSA of 71,038 global SARS-CoV-2 genomes is performed using MAFFT, which is followed by their phylogenetic analysis using Nextstrain. The corresponding phylogenetic tree is shown in Figure 1B. MAFFT merges local and global algorithms for MSA, and it uses two different heuristic methods such as progressive (FFT-NS-2) and iterative refinement (FFT-NS-i). To create a provisional MSA, FFT-NS-2 calculates all-pairwise distances from which refined distances are calculated. Thereafter, FFT-NS-i is performed to get the final MSA. As MAFFT uses fast Fourier transform, it scores over other alignment techniques. On the other hand, Nextstrain is a collection of open-source tools, which is useful for understanding the evolution and spread of pathogen, particularly during an outbreak. By taking advantage of this tool, in this work, the evolution and geographic distribution of SARS-CoV-2 genomes are visualized by creating the metadata in our High Performance Computing environment.

Once the alignment and the phylogenetic analysis are completed, hotspot mutations as deletions and substitutions are identified in the coding regions based on entropy greater than or equal to 0.3. Furthermore, 10,286 Indian sequences are considered as an example to identify such mutations as well. The corresponding phylogenetic tree for Indian sequences is shown in Figure 1C. Moreover, using the codon table, amino acid changes in the SARS-CoV-2 proteins for the corresponding mutations are highlighted as well. The hotspot mutations are identified considering their entropy values, which are calculated as:

$$E=\ln\;5+\sum \overset{\delta }{\underset{\gamma }{\lambda }}\;\;\left[\;\ln\;\left(\lambda_{\gamma }^{\delta }\right)\;\right]$$ (1)

where $\lambda_{\gamma }^{\delta }$ represents the frequency of each residue *γ* occurring at position *δ* and 5 represents the four possible residues as nucleotides plus gap. Thereafter, the amino acid changes in the SARS-CoV-2 proteins for the non-synonymous deletions and substitutions for both global and Indian sequences are graphically visualized as shown in Figure 1D. Finally, these changes are also used for the evaluation of their functional characteristics and are visualized in the respective protein structure as well.

## 3 Results

**FIGURE 2:**
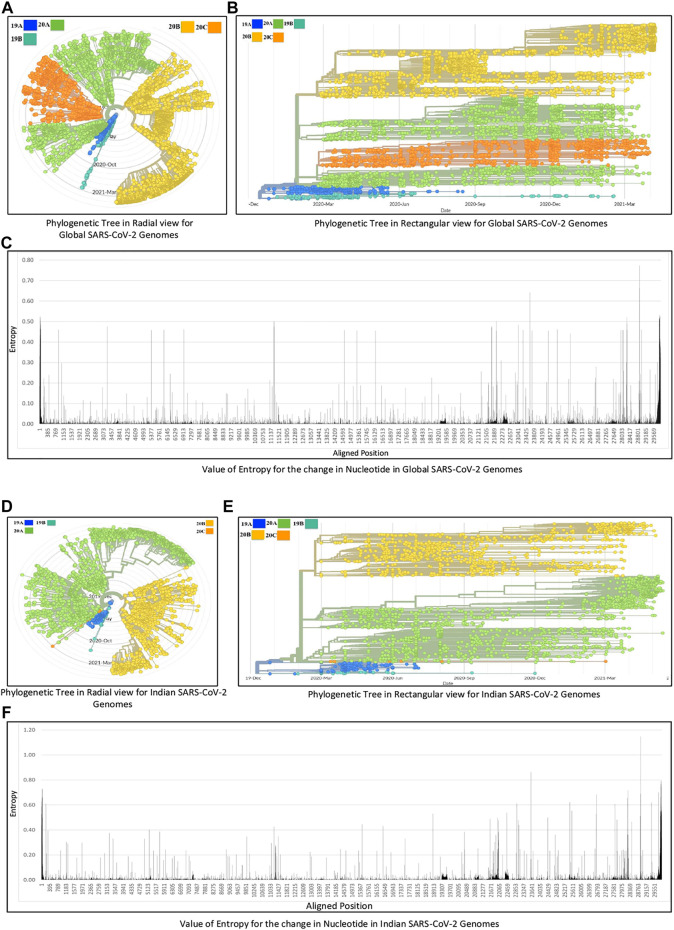
Phylogenetic analysis of **(A, B, C)** global and **(D, E, F)** Indian SARS-CoV-2 genomes.

The experiments in this work are carried out according to the pipeline as given in Figure 1A. Initially, MSA of 71,038 global SARS-CoV-2 genomes across 98 countries is carried out using MAFFT followed by their phylogenetic analysis using Nextstrain, which revealed five clades: 19A, 19B, 20A, 20B, and 20C. The number of sequences for each country is reported in Supplementary Table S1. This resulted in the identification of hotspot mutation points as deletions and substitutions in the coding regions based on entropy. In this regard, only those hotspot mutations are considered whose entropy values are greater than or equal to 0.3. The entropy values for each of the genomic coordinates for both global and Indian sequences are provided in Supplementary Table S2. The mutation statistics by considering different threshold values of entropy for each category are reported in Table 1. Based on the results in this table, the entropy value of 0.3 is considered as the threshold for choosing the hotspot mutations. It is to be noted that choosing a threshold value as either 0.2 or 0.1 will lead to a huge amount of hotspot mutations, which is not desired. As a consequence of choosing entropy threshold of 0.3, 45 unique hotspot mutations are identified, which resulted in 39 non-synonymous deletions and substitutions with nine unique deletions and 22 unique amino acid changes. Also, out of the 98 countries that are considered for global analysis, India with 10,286 sequences is taken as an example to demonstrate the mutations for a particular country as well. In this regard, 52 unique hotspot mutations provide 45 non-synonymous deletions and substitutions with five unique amino acid changes for deletions and 36 unique amino acid changes for substitutions. The analysis on other countries with the most number of sequences is provided in the Supplementary Material. The phylogenetic trees in radial and rectangular views considering global analysis are shown in Figures 2A,B, respectively, while for Indian sequences, such views are provided in Figures 2D,E, respectively. These phylogenetic trees respectively show the evolution of the global and Indian SARS-CoV-2 genomes over the months. For the benefit of the readers, it is important to mention that the number of sequences does not have any direct relationship with the number of hotspot mutations. The number of hotspots is based on the entropy value, which in turn depends on the frequency of mutations at a given genomic coordinate. So even with smaller number of sequences, if the frequency of mutations is higher than that with larger number of sequences, it will produce more hotspot mutations. Thus, with 71,038 global sequences, 45 unique hotspot mutations are identified, while for 10,286 Indian sequences, 52 such mutations are identified.

**TABLE 1:** Mutation statistics of 71,038 global and 10,286 Indian SARS-CoV-2 genomes by considering different threshold values.

Threshold value	Coding regions of global SARS-CoV-2 genomes																									
	NSP1	NSP2	NSP3	NSP4	3CL-Pro	NSP6	NSP7	NSP8	NSP9	NSP10	NSP11	RdRp	Helicase	Exon	endoRNAse	NSP16	Spike	ORF3a	Envelope	Membrane	ORF6	ORF7a	ORF7b	ORF8	Nucleocapsid	ORF10
> =0.60	0	0	0	0	0	0	0	0	0	0	0	0	0	0	0	0	1	0	0	0	0	0	0	0	3	0
> =0.50 to < 0.60	0	0	0	0	0	1	0	0	0	0	0	0	0	0	0	0	1	0	0	0	0	0	0	0	0	0
> =0.40 to < 0.50	0	1	4	0	0	8	0	0	0	0	0	3	0	0	0	0	13	1	0	0	0	0	0	3	4	0
> =0.30 to < 0.40	0	0	0	0	0	0	0	0	0	0	0	0	0	0	0	0	1	0	0	0	0	0	0	1	0	0
> =0.20 to < 0.30	1	1	1	0	0	1	0	0	0	0	0	0	0	1	0	1	6	1	0	3	0	2	0	1	3	1
> =0.10 to < 0.20	1	3	7	4	1	3	0	0	0	0	0	6	3	3	1	1	14	1	0	0	0	0	1	7	8	0
> =0.05 to < 0.10	1	3	18	3	2	2	0	0	3	0	0	5	6	2	4	1	25	7	0	2	1	2	0	2	5	0
**Threshold value**	**Coding regions of Indian SARS-CoV-2 genomes**																									
> =0.60	0	0	0	0	0	0	0	0	0	0	0	0	0	0	0	0	2	1	0	1	0	1	0	1	4	0
> =0.50 to < 0.60	0	0	0	0	0	0	0	0	0	0	0	0	0	1	0	0	1	1	0	1	0	0	0	0	1	0
> =0.40 to < 0.50	0	0	1	0	0	1	0	0	0	0	0	1	1	0	0	0	9	0	0	0	0	1	0	1	1	0
> =0.30 to < 0.40	1	1	4	1	0	1	0	0	0	0	0	0	1	0	1	0	6	0	0	0	0	0	0	4	1	0
> =0.20 to < 0.30	0	3	4	3	0	5	0	0	1	0	0	4	1	0	2	1	16	1	0	1	0	1	0	4	4	0
> =0.10 to < 0.20	0	1	12	5	0	10	0	0	1	0	0	3	2	3	1	1	8	2	0	0	2	0	1	2	4	0
> =0.05 to < 0.10	0	7	11	4	1	5	0	1	1	0	0	4	1	8	1	3	53	3	0	11	0	0	0	5	7	1

The list of hotspot mutations for the global and Indian SARS-CoV-2 genomes along with their associated details is respectively provided in Tables 2 and 3. For example, in Table 2, genomic coordinate 28,881 in Nucleocapsid with nucleotide changes G > A and G > T has the highest entropy value of 0.773655. India also shows the same mutation but with an entropy value of 1.14807 as shown in Table 3. Please note that mutations like G28881A and G28883C may have an impact on antigenicity of Nucleocapsid protein (Yuan et al., 2020). The entropy values for the corresponding nucleotide changes for global analysis are shown in Figure 2C, while for India, the same is shown in Figure 2F. It is to be noted that the total number of unique amino acid changes for deletions and substitutions is less than the number of non-synonymous deletions and substitutions. One of the reasons for this can be that if there are deletions at consecutive genomic coordinates, the corresponding amino acid changes are the same. For example, as can be seen from Table 2, at the three consecutive genomic coordinates 11,288, 11,289, and 11,290, deletion has occurred with the amino acid change as S106-. Thus, though the number of non-synonymous deletions is 3, the number of unique amino acid change is 1. This is true for other such changes as well.

**TABLE 2:** List of hotspot mutations for 71,038 global SARS-CoV-2 genomes along with the protein change.

Genomic coordinate	Overall entropy	Nucleotide change	Amino acid change	Protein coordinate	Gene
28,881	0.773655	G > A, G > T	R > K, R > M	203	Nucleocapsid
28,883	0.663399	G > C	G > R	204	Nucleocapsid
28,882	0.663308	G > A	R > R	203	Nucleocapsid
23,604	0.642160	C > A, C > G	P > H, P > R	681	Spike
11,296	0.502171	T > -	F > -	108	NSP6
21,993	0.500865	A > -	Y > -	144	Spike
11,291	0.499603	G > -	G > -	107	NSP6
28,280	0.491543	G > C	D > H	3	Nucleocapsid
23,063	0.484066	A > T	N > Y	501	Spike
21,770	0.476393	G > -	V > -	70	Spike
3,267	0.475810	C > T	T > I	183	NSP3
11,288	0.474924	T > -	S > -	106	NSP6
11,289	0.472836	C > -	S > -	106	NSP6
21,765	0.471435	T > -	I > -	68	Spike
21,767	0.469881	C > -	H > -	69	Spike
11,290	0.467890	T > -	S > -	106	NSP6
21,766	0.467479	A > -	I > -	68	Spike
21,768	0.467116	A > -	H > -	69	Spike
21,769	0.466151	T > -	H > -	69	Spike
11,293	0.465319	T > -	G > -	107	NSP6
11,292	0.464056	G > -	G > -	107	NSP6
11,294	0.463926	T > -	F > -	108	NSP6
24,914	0.461770	G > C	D > H	1118	Spike
6,954	0.461746	T > C	I > T	1412	NSP3
28,977	0.460661	C > T	S > F	235	Nucleocapsid
21,992	0.460243	T > -	Y > -	144	Spike
913	0.460233	C > T	S > S	36	NSP2
11,295	0.459624	T > -	F > -	108	NSP6
5,986	0.459543	C > T	F > F	1089	NSP3
28,282	0.459253	T > A	D > E	3	Nucleocapsid
28,048	0.458864	G > T	R > I	52	ORF8
14,676	0.458373	C > T	P > P	412	RdRp
23,271	0.458086	C > A	A > D	570	Spike
28,281	0.458038	A > T	D > V	3	Nucleocapsid
27,972	0.457841	C > T	Q > *	27	ORF8
5,388	0.457761	C > A	A > D	890	NSP3
28,111	0.457624	A > G	Y > C	73	ORF8
23,709	0.456643	C > T	T > I	716	Spike
24,506	0.455921	T > G	S > A	982	Spike
15,279	0.455884	C > T	H > H	613	RdRp
16,176	0.455573	T > C	T > T	912	RdRp
21,991	0.455314	T > -	V > -	143	Spike
25,563	0.442049	G > T	Q > H	57	ORF3a
22,227	0.310063	C > T	A > V	222	Spike
28,253	0.300528	C > T, C > -	F > F, F > -	120	ORF8

**TABLE 3:** List of hotspot mutations for 10,286 Indian SARS-CoV-2 genomes along with the protein change.

Genomic coordinate	Overall entropy	Nucleotide change	Amino acid change	Protein coordinate	Gene
28,881	1.14807	G > A, G > T	R > K, R > M	203	Nucleocapsid
23,604	0.8631	C > A, C > G	P > H, P > R	681	Spike
28,882	0.69019	G > A	R > R	203	Nucleocapsid
28,883	0.68846	G > C	G > R	204	Nucleocapsid
26,767	0.68419	T > C, T > G	I > T, I > S	82	Membrane
28,253	0.65534	C > T, C > -	F > F, F > -	120	ORF8
25,469	0.6227	C > T	S > L	26	ORF3a
29,402	0.61955	G > T	D > Y	377	Nucleocapsid
22,917	0.61006	T > G	L > R	452	Spike
27,638	0.60866	T > C	V > A	82	ORF7a
25,563	0.55354	G > T	Q > H	57	ORF3a
22,444	0.53665	C > T	D > D	249	Spike
18,877	0.52834	C > T	L > L	280	Exon
26,735	0.52715	C > T	Y > Y	71	Membrane
28,854	0.51198	C > T	S > L	194	Nucleocapsid
24,410	0.49845	G > A	D > N	950	Spike
21,987	0.49717	G > A	G > D	142	Spike
21,618	0.48836	C > G	T > R	19	Spike
27,752	0.48264	C > T	T > I	120	ORF7a
22,034	0.47915	A > -	R > -	158	Spike
22,995	0.47879	C > A	T > K	478	Spike
28,461	0.46436	A > G	D > G	63	Nucleocapsid
15,451	0.44421	G > A	G > S	671	RdRp
23,012	0.44086	G > C	E > Q	484	Spike
22,033	0.4385	C > -	F > -	157	Spike
16,466	0.43082	C > T	P > L	77	Helicase
22,032	0.42673	T > -	F > -	157	Spike
11,201	0.42554	A > G	T > A	77	NSP6
28,249	0.41704	A > -	D > -	119	ORF8
5,184	0.40139	C > T	P > L	822	NSP3
22,031	0.40074	T > -	F > -	157	Spike
313	0.39475	C > T	L > L	16	NSP1
22,029	0.38676	A > -	E > -	156	Spike
5,700	0.38604	C > A	A > D	994	NSP3
20,396	0.38407	A > G	K > R	259	endoRNAse
3,267	0.37579	C > T	T > I	183	NSP3
22,030	0.3738	G > -	E > -	156	Spike
28,251	0.36694	T > -	F > -	120	ORF8
28,248	0.36497	G > -	D > -	119	ORF8
24,775	0.36197	A > T	Q > H	1071	Spike
21,895	0.35931	T > C	D > D	111	Spike
28,280	0.35905	G > C	D > H	3	Nucleocapsid
28,250	0.35546	T > -	D > -	119	ORF8
28,252	0.351	T > -	F > -	120	ORF8
11,418	0.34861	T > C	V > A	149	NSP6
9,891	0.34766	C > T	A > V	446	NSP4
17,523	0.33196	G > T	M > I	429	Helicase
3,457	0.3314	C > T	Y > Y	246	NSP3
4,965	0.32981	C > T	T > I	749	NSP3
22,022	0.31618	G > A	E > K	154	Spike
1191	0.30404	C > T	P > L	129	NSP2
21,846	0.30253	C > T	T > I	95	Spike

**FIGURE 3:**
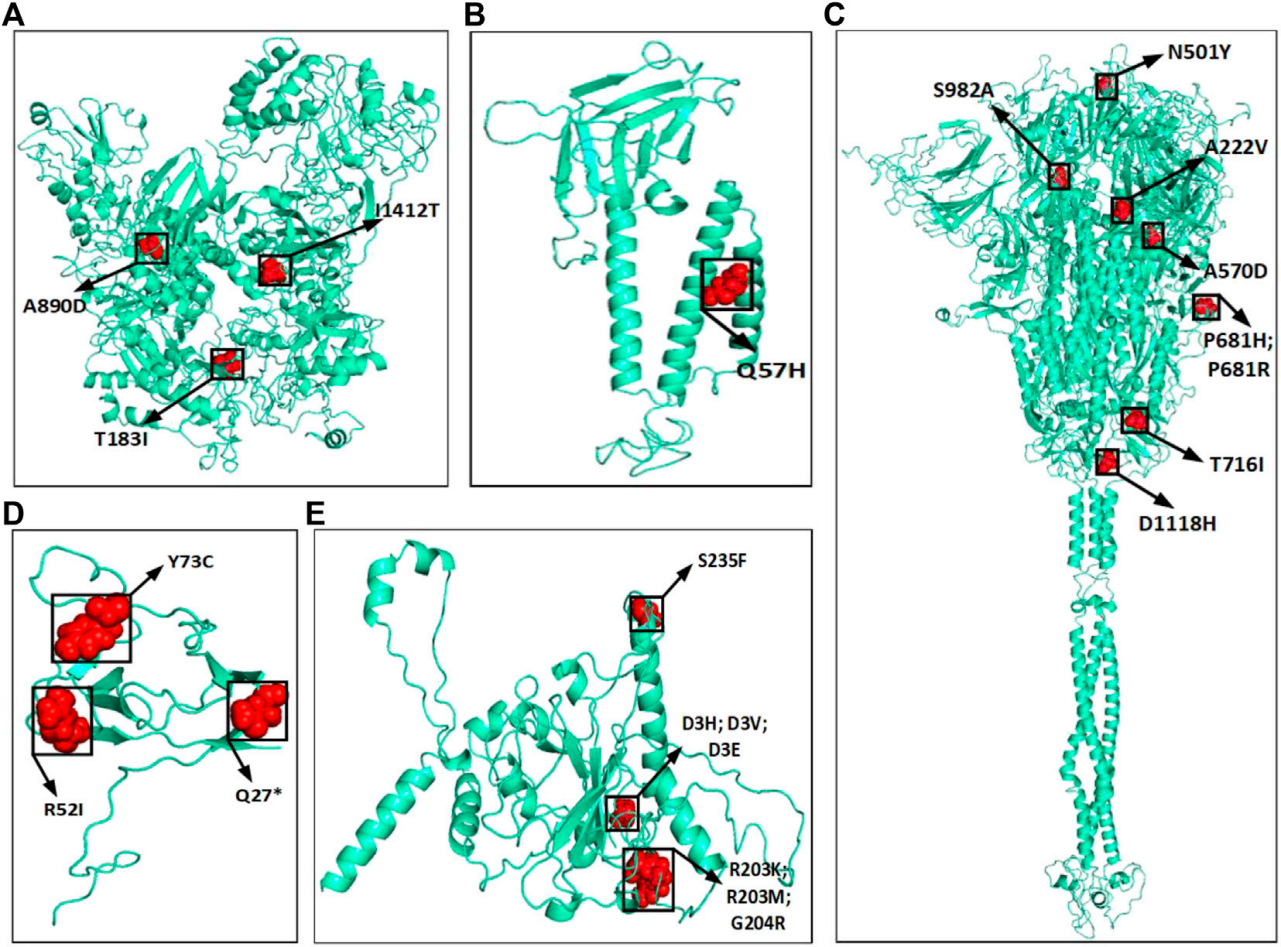
Highlighted amino acid changes in the protein structures for the non-synonymous substitutions or missense hotspot mutations for global SARS-CoV-2 genomes in **(A)** NSP3, **(B)** ORF3a, **(C)** Spike, **(D)** ORF8, and **(E)** Nucleocapsid.

**FIGURE 4:**
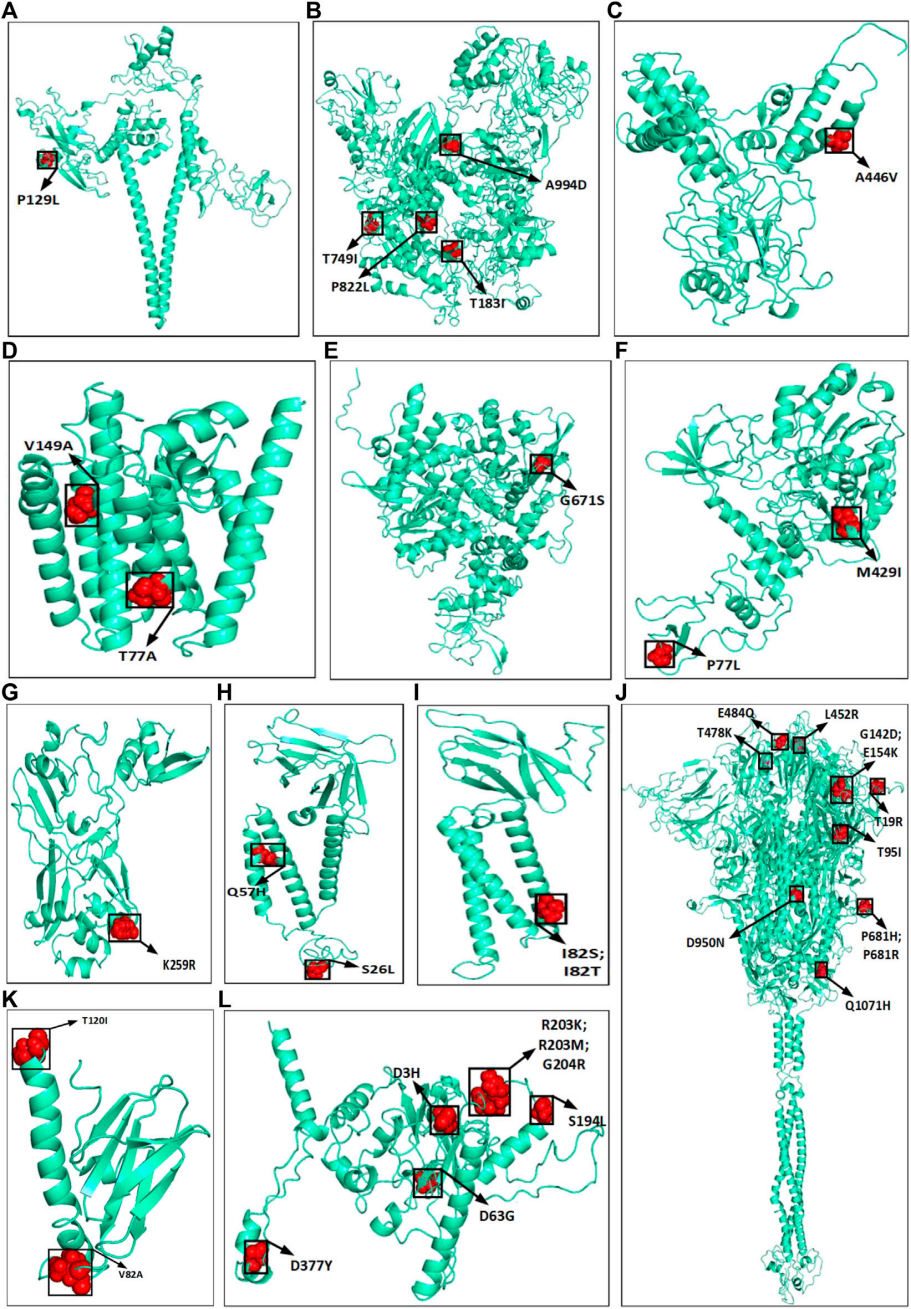
Highlighted amino acid changes in the protein structures for the non-synonymous substitutions or missense hotspot mutations for Indian SARS-CoV-2 genomes in **(A)** NSP2, **(B)** NSP3, **(C)** NSP4, **(D)** NSP6, **(E)** RdRp, **(F)** helicase, **(G)** endoRNAse, **(H)** ORF3a, **(I)** Membrane, **(J)** Spike, **(K)** ORF7a, and **(L)** Nucleocapsid.

**FIGURE 5:**
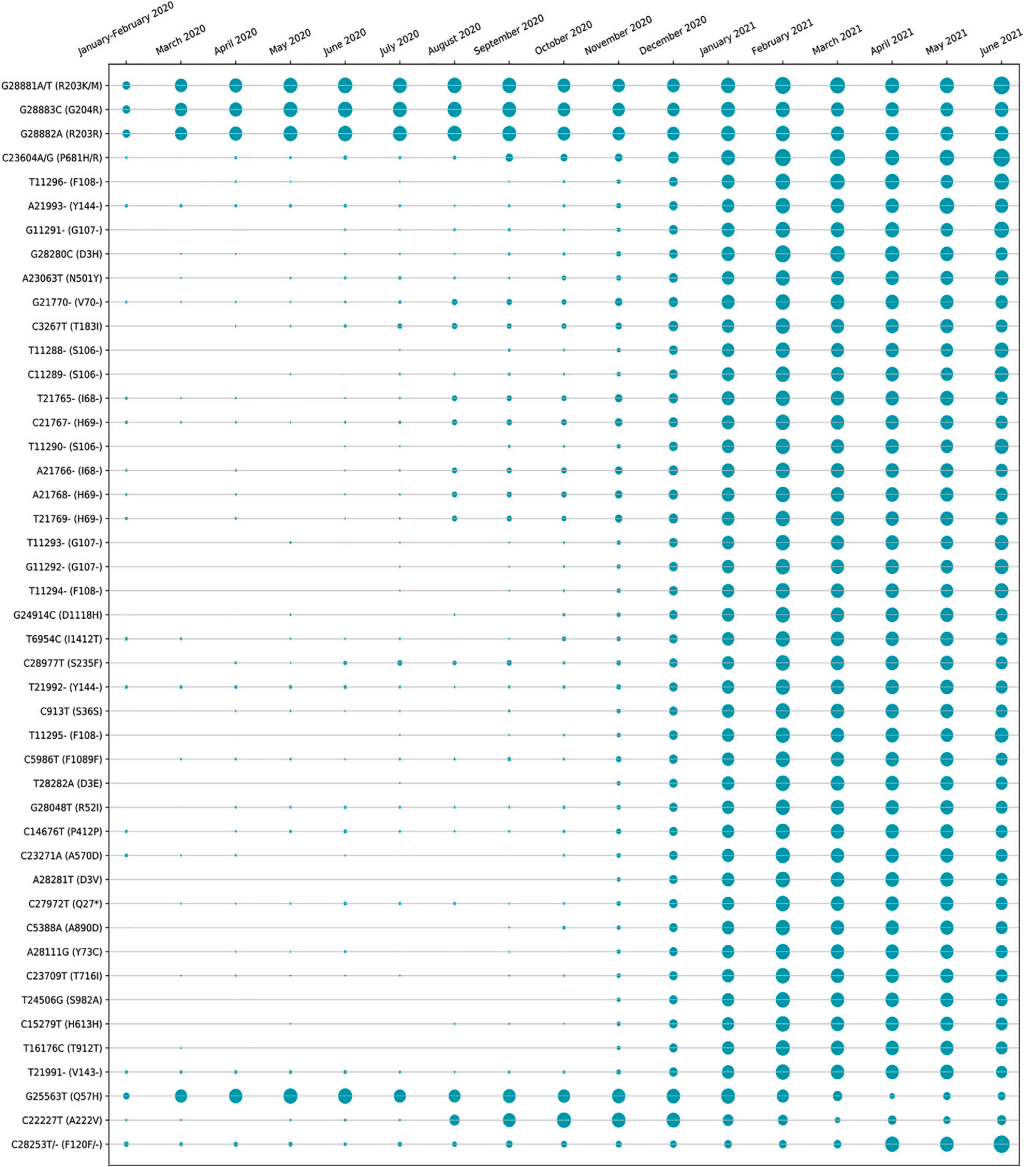
Month-wise evolution of global SARS-CoV-2 genomes based on entropy.

**FIGURE 6:**
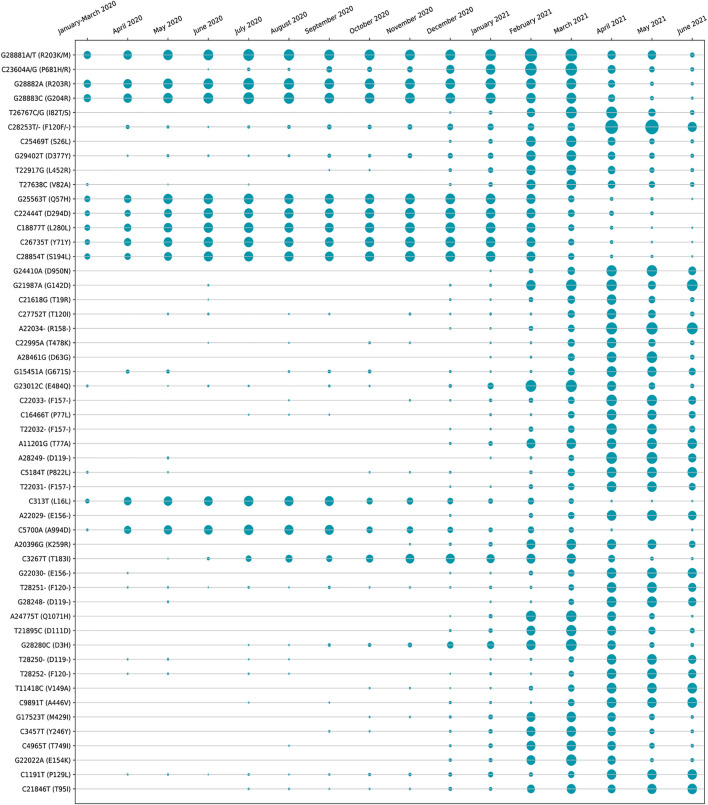
Month-wise evolution of Indian SARS-CoV-2 genomes based on entropy.

**FIGURE 7:**
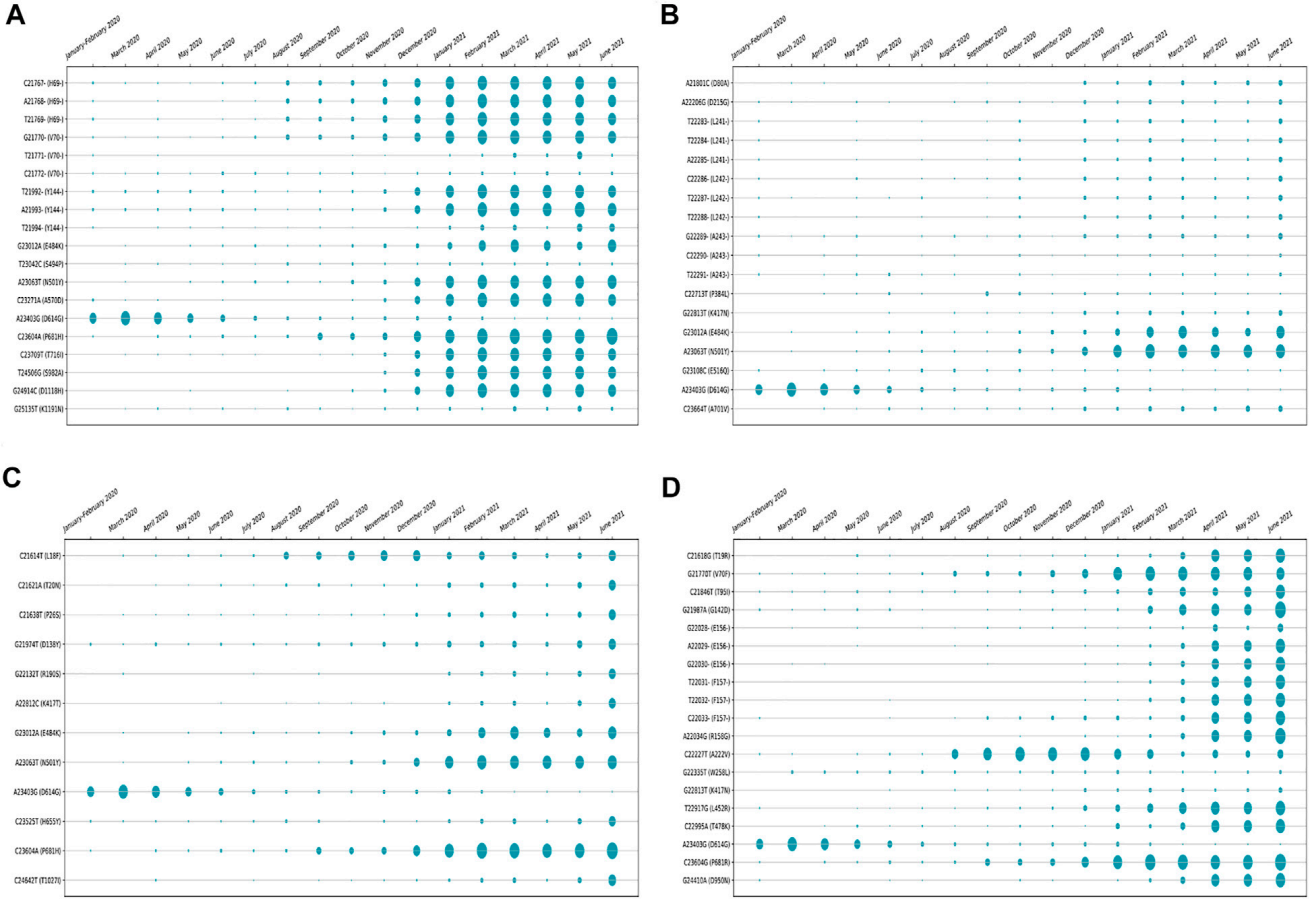
Month-wise evolution of **(A)** Alpha (B.1.1.7), **(B)** Beta (B.1.351), **(C)** Gamma (501.V3), and **(D)** Delta (B.1.617.2) variants in global SARS-CoV-2 genomes.

The amino acid changes in protein for the non-synonymous deletions and substitutions as reported in Tables 2 and 3 are visualized in Figure 1D; Supplementary Figure S1. All the amino acid changes in the protein for the non-synonymous substitutions or missense mutations for the global sequences are shown in Figure 3, while the same for the Indian sequences are depicted in Figure 4. The month-wise virus evolution in terms of entropy for both global and Indian genomic sequences is visualized respectively in Figures 5 and 6, while the corresponding entropy values are reported in Supplementary Tables S3 and S4. For example, it can be seen from both the figures that both P681H and P681R, which are part of the variant of concerns Alpha or B.1.1.7 and Delta or B.1.617.2, have evolved over time globally and for India as well. It is to be noted that due to the lack of appropriate number of sequences, the data of January and February 2020 have been merged for the global analysis, while for India, such merging is for the months January to March 2020. Also, please note that since the calculation of entropy is performed on aligned sequences, only coding regions are considered for the identification of hotspot mutations, as the non-coding regions exhibit high entropy values and can be misleading while selecting such mutation points as hotspot mutations. Furthermore, the evolution of the mutation points for global SARS-CoV-2 genomes pertaining to the different variants of concern like Alpha, Beta, Gamma, and Delta as declared by the WHO is also reported respectively in Figures 7A,B,C,D. It can be observed from the figures that the popular mutation D614G, which is common in all the variants though predominant in the earlier months of the pandemic, has waned over time. Also, the mutation T478K, which is unique to the Delta variant, is known to facilitate antibody escape (Planas et al., 2021). Some important hotspot mutations like H69-, V70-, Y144-, A222V, N501Y, A570D, P681H, and P681R identified in this study are associated with the different SARS-CoV-2 variants of concern like Alpha, Beta, Gamma, and Delta.

**FIGURE 8:**
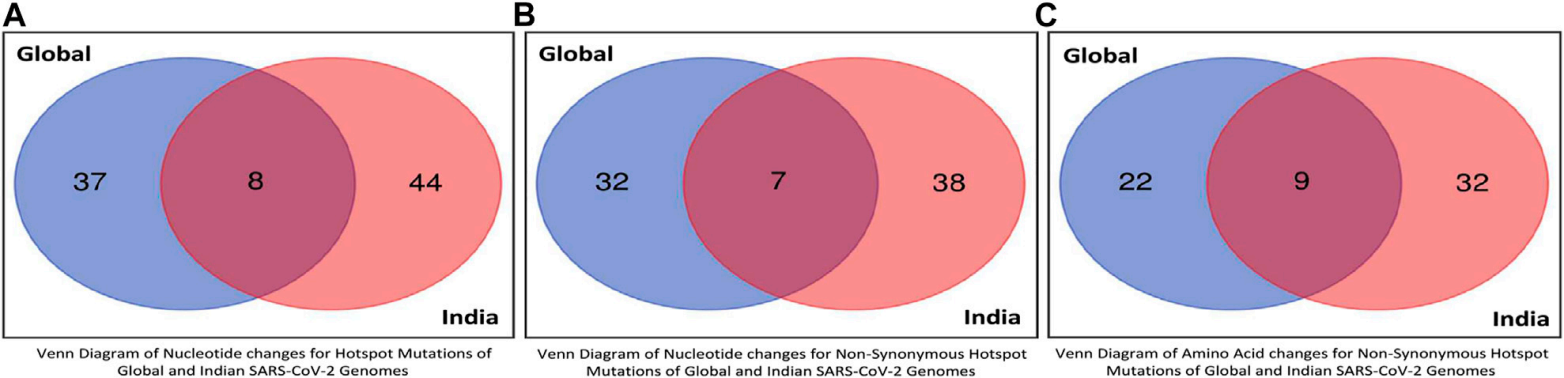
Venn diagrams of global and Indian SARS-CoV-2 genomes to represent common hotspot mutations.

The unique and common hotspot mutations between global and Indian sequences are represented in the form of Venn diagram in Figures 8A,B, which shows the unique and common non-synonymous hotspot mutations, while the unique and common amino acid changes are shown in Figure 8C. As shown in Figure 8A, there are 37 and 44 unique mutations in global and Indian sequences, while eight are common in both. For non-synonymous hotspot deletions and substitutions, there are 32 and 38 unique mutations in each category, while the common number of such mutations is seven as reported in Figure 8B. For amino acid changes, as shown in Figure 8C, these statistics are 22, 32, and nine. The Venn diagram showing the common and unique hotspot mutations for global and Indian sequences with Alpha, Beta, Gamma, and Delta variants of SARS-CoV-2 is reported in Supplementary Figure S2. For example, in Supplementary Figure S2A, there are four unique mutations in both global sequences and Alpha variant, while there are nine mutations that are common to both.

## 4 Discussion

There are spurts of new waves in almost every country around the globe. India has already gone through the massively catastrophic second wave, and according to the experts, a third wave is imminent. This can be attributed to the fact that the virus is evolving and new strains are getting identified, thereby making the study of this ever-evolving virus all the more important. The functional characteristics of some important mutations in the global and Indian SARS-CoV-2 genomic sequences are reported in Table 4.

**TABLE 4:** Functional characteristics of some important mutations.

Mutations	Functional characteristics
H69-	Leads to conformational changes in Spike protein (Meng et al., 2021; McCarthy et al., 2021)
V70-	Leads to conformational changes in Spike protein (Meng et al., 2021; McCarthy et al., 2021)
Y144-	Reduces affinity of antibody binding (McCarthy et al., 2021)
L452R	Increases the binding ability of the ACE2 receptor and can also reduce the attaching capability of vaccine-simulated antibodies with Spike protein (Garcia-Beltran et al., 2021)
T478K	Facilitates antibody escape (Planas et al., 2021)
E484Q	Associated with reduced sera neutralization (Greaney et al., 2021)
N501Y	Highest binding affinity with human receptor cell hACE2 and resistant to neutralization (Luan et al., 2021)
P681H	Near furin cleavage site, may affect transmissibility of the virus (Boehm et al., 2021)
P681R	Near furin cleavage site, may affect transmissibility of the virus (Boehm et al., 2021)

Structural changes in amino acid residues may sometimes lead to functional instability in proteins due to change in protein translations. To judge their characteristics, these changes are demonstrated through sequence and structural homology-based prediction for the hotspot deletions and missense mutations for global and Indian sequences in Table 5. The tools used for these predictions are PolyPhen-2 (Polymorphism Phenotyping) (Adzhubei et al., 2010) and I-Mutant 2.0 (Capriotti et al., 2005). PolyPhen-2^4^ works with sequence, structural, and phylogenetic information of missense mutations, while I-Mutant 2.0^5^ uses support vector machine (SVM) for the automatic prediction of protein stability changes upon missense mutations. PolyPhen-2 is used to find the damaging hotspot mutations, and I-Mutant 2.0 determines protein stability. To determine if a mutation is damaging using PolyPhen-2, its score is considered, which lies between 0 and 1. If the score is close to 1, then a mutation is considered to be damaging. It can be concluded from Table 5 that out of the 22 unique amino acid changes for substitutions in global sequences, 14 are damaging, while for Indian sequences, 24 are damaging out of 36 changes. It is important to note that in case of protein, damaging mostly defines instability. Generally, this is used for human proteins. As a consequence, if the human protein is damaging in nature because of mutations, then the human protein–protein interactions may occur with high or low binding affinity. Now in case of virus, similar consequences may happen, which means that if the virus protein is damaged because of mutations, it may interact with human proteins with similar binding affinity. As a result, the virus may acquire characteristics like transmissibility and escaping antibodies (Alenquer et al., 2021; Harvey et al., 2021).

**TABLE 5:** Sequence and structural homology-based prediction of non-synonymous substitution as hotspot mutations along with their protein structural stability for 71,038 global SARS-CoV-2 genomes.

Change in	Change in	Mapped with	PolyPhen-2		I-mutant 2.0	
Nucleotide	Amino acid	Coding regions	Prediction	Score	Stability	DDG (kcal/mol)
G28881A	R203 K	Nucleocapsid	Probably damaging	0.969	Decrease	−2.26
G28881T	R203M	Nucleocapsid	Probably damaging	0.998	Decrease	−1.52
G28883C	G204R	Nucleocapsid	Probably damaging	1	No change	0
C23604A	P681H	Spike	Not generated	Not generated	Decrease	−0.92
C23604G	P681R	Spike	Not generated	Not generated	Decrease	−0.79
G28280C	D3H	Nucleocapsid	Probably damaging	1	Increase	0.34
A23063T	N501Y	Spike	Benign	0.145	Decrease	−0.34
C3267T	T183I	NSP3	Not generated	Not generated	Decrease	-0.1
G24914C	D1118H	Spike	Probably damaging	0.998	Decrease	−0.1
T6954C	I1412T	NSP3	Benign	0.026	Decrease	−2.78
C28977T	S235F	Nucleocapsid	Probably damaging	0.998	Increase	2.43
T28282A	D3E	Nucleocapsid	Probably damaging	0.997	Decrease	−0.02
G28048T	R52I	ORF8	Probably damaging	1	Decrease	−0.09
C23271A	A570D	Spike	Benign	0.031	Decrease	−1.32
A28281T	D3V	Nucleocapsid	Probably damaging	1	Decrease	−0.22
C5388A	A890D	NSP3	Probably damaging	1	Decrease	−1.09
A28111G	Y73C	ORF8	Probably damaging	0.994	Increase	1.04
C23709T	T716I	Spike	Possibly damaging	0.696	Decrease	−0.95
T24506G	S982A	Spike	Probably damaging	0.996	Decrease	−1.36
C22227T	A222V	Spike	Benign	0.001	Increase	0.48
T26767G	I82S	Membrane	Possibly damaging	0.951	Decrease	−2
C25469T	S26L	ORF3a	Benign	0.017	Increase	0.92
G29402T	D377Y	Nucleocapsid	Probably damaging	1	Increase	0.51
T22917G	L452R	Spike	Benign	0.04	Decrease	−1.4
T27638C	V82A	ORF7a	Possibly damaging	0.732	Decrease	-2.18
G25563T	Q57H	ORF3a	Probably damaging	0.983	Decrease	−1.12
C28854T	S194L	Nucleocapsid	Probably damaging	0.994	Increase	0.45
G24410A	D950N	Spike	Possibly damaging	0.731	Increase	0.15
G21987A	G142D	Spike	Benign	0.051	Decrease	−1.17
C21618G	T19R	Spike	Benign	0.004	Decrease	−0.12
C27752T	T120I	ORF7a	Possibly damaging	0.915	Decrease	−0.26
C22995A	T478K	Spike	Benign	0	Decrease	−0.09
A28461G	D63G	Nucleocapsid	Benign	0	Decrease	−0.57
G15451A	G671S	RdRp	Probably damaging	1	Decrease	−0.29
G23012C	E484Q	Spike	Possibly damaging	0.786	Decrease	−0.48
C16466T	P77L	Helicase	Probably damaging	1	Decrease	−1.03
A11201G	T77A	NSP6	Possibly damaging	0.577	Decrease	−0.7
C5184T	P822L	NSP3	Benign	0.007	Decrease	−0.54
C5700A	A994D	NSP3	Probably damaging	0.972	Decrease	−0.78
A20396G	K259R	endoRNAse	Benign	0	Decrease	−0.49
A24775T	Q1071H	Spike	Possibly damaging	0.998	Decrease	−1.19
T11418C	V149A	NSP6	Possibly damaging	0.865	Decrease	−3.43
C9891T	A446V	NSP4	Probably damaging	0.999	Increase	0.64
G17523T	M429I	Helicase	Possibly damaging	0.649	Decrease	−1.26
C4965T	T749I	NSP3	Probably damaging	0.996	Decrease	−0.92
G22022A	E154 K	Spike	Not generated	Not Generated	Decrease	−1.4
C1191T	P129L	NSP2	Possibly damaging	0.888	Decrease	−0.53
C21846T	T95I	Spike	Probably damaging	0.999	Decrease	−1.8

**FIGURE 9:**
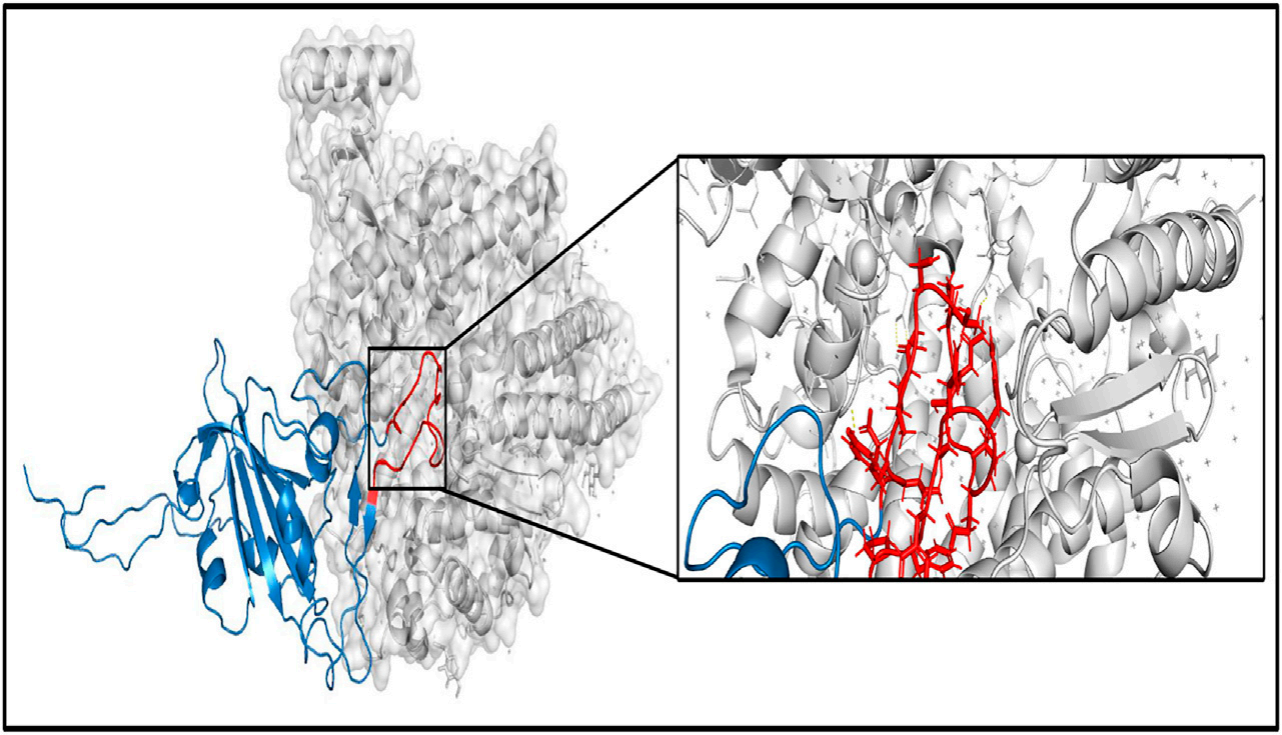
Binding between RBD region of Spike protein (specifically 471–492 the region marked in red)) and human ACE2 protein. RBD, receptor-binding domain.

Another important parameter to judge the functional and structural activities of a protein is protein stability, which dictates the conformational structure of a protein. Any change in protein stability may cause misfolding, degradation, or aberrant conglomeration of proteins. I-Mutant 2.0 uses free energy change values (DDG (kcal/mol)) to predict the changes in the protein stability wherein a negative value of DDG indicates that the protein has a decreasing stability, while a positive value indicates an increase in stability. For example, the very low DDG value of G25563T shows that there is a decreased protein stability, thereby resulting in a reduction of virus virulence (Cheng et al., 2021). The results from I-mutant 2.0 show that out of the 14 and 24 unique damaging changes for global and Indian sequences, 10 and 18 changes respectively decrease the stability of the protein structures. Figure 9 shows the binding affinity between the RBD of Spike protein and human ACE2 protein performed using SSIPe^6^ (Huang et al., 2019) for the four mutations of SARS-CoV-2, viz., L452R, T478K, E484Q, and N501Y, taking place in such domain. The region marked in red shows the exact positions (471–492) where the binding takes place. To report the binding affinity using SSIPe, initially the RBD region of Spike protein (Woo et al., 2020) is docked with human ACE2 protein^7^ using PatchDock^8^. The best docked structure is then provided as an input to SSIPe. Table 6 further reports the binding affinity values for the four mutations. A strongly favorable mutation is usually defined as the one that has DDG value ≤ −1.5 kcal/mol, while a strongly unfavorable mutation is the one that has DDG value ≥1.5 kcal/mol. The DDG value of −0.769 kcal/mol for E484Q indicates that this is a favorable mutation, while DDG values of 1.083, 1.248, and 0.236 kcal/mol for L452R, T478K, and N501Y indicate that these mutations are somewhat unfavorable. These results corroborate our earlier explanation that because of mutation, virus–human protein–protein interactions may occur with high or low binding affinity.

**TABLE 6:** Binding affinity of the mutations in RBD region of Spike protein and human ACE2 protein.

Genomic coordinate	Nucleotide change	Amino acid change	Protein coordinate	DDG (kcal/mol)	SSIPscore	EvoEFscore
22,917	T > G	L > R	452	1.083	2.083	−1.91
22,995	C > A	T > K	478	1.248	1.779	−0.77
23,012	G > C	E > Q	484	−0.769	1.098	−5.22
23,063	A > T	N > Y	501	0.236	0	0.09

Note. RBD, receptor-binding domain.

Supplementary Figure S3 shows the percentage of nucleotide change and frequency of nucleotide change for hotspot mutations for global and Indian sequences. For example, in Supplementary Figure S3A, the occurrence of nucleotide change G > A in 71,038 global sequences is almost 45%, while the number of times it occurs in 45 hotspot mutations is two, as is also evident from Table 2. It can also be seen from Supplementary Figures S3B, S3D that 10 and 16 out of 39 and 45 non-synonymous mutations are from C to T, thereby representing abundant transition. This transition increases the frequency of codons for hydrophobic amino acids and provides evidence of potential antiviral editing mechanisms driven by host (Yuan et al., 2020). Also, more C-to-T transition means less CpG abundance, indicating rapid adaptation of virus in host. This CpG deficiency, which leads to evasion of host antiviral defense mechanisms, is exhibited the most in SARS-CoV-2 virus (Xia, 2020).

## 5 Conclusion

With the imminent third wave, it is very crucial to understand the evolution of SARS-CoV-2. In this regard, MSA of 71,038 SARS-CoV-2 genomes of 98 countries over the period from January 2020 to June 2021 is performed using MAFFT followed by phylogenetic analysis to visualize the evolution of SARS-CoV-2. This resulted in the identification of hotspot mutations as deletions and substitutions in the coding regions based on entropy, which should be greater than or equal to 0.3. Consequently, a total of 45 unique hotspot mutations out of which 39 non-synonymous deletions and substitutions are identified with nine unique amino acid changes for deletions and 22 unique amino acid changes for substitutions. Moreover, 10,286 Indian sequences are considered from 71,038 global SARS-CoV-2 sequences as a demonstrative example, which gives 52 unique hotspot mutations, resulting in 45 non-synonymous deletions and substitutions with five unique amino acid changes for deletions and 36 unique amino acid changes for substitutions. Some important mutations in such sequences pertaining to the Delta variant of SARS-CoV-2 are T19R, G142D, E156-, F157-, L452R, T478K, and P681R. Furthermore, the evolution of the hotspot mutations along with the mutations in variants of concern is visualized, and their characteristics are also discussed. Moreover, for all the missense mutations, the functional consequences of amino acid changes in the respective protein structures are calculated using PolyPhen-2 and I-Mutant 2.0. Finally, SSIPe is used to report the binding affinity between the RBD of Spike protein and human ACE2 protein by considering L452R, T478K, E484Q, and N501Y hotspot mutations in that region.

## Data Availability

The aligned 71038 Global SARS-CoV-2 genomes with the reference sequence and the final results of this work are available at http://www.nitttrkol.ac.in/indrajit/projects/COVID-Hotspot-Mutation-Global-71K/. Further inquiries can be directed to the corresponding author.

## Footnotes

1
https://www.gisaid.org/

2
https://www.ncbi.nlm.nih.gov/nuccore/1798174254

3
https://zhanglab.ccmb.med.umich.edu/COVID-19/

4
http://genetics.bwh.harvard.edu/pph2/

5
https://folding.biofold.org/i-mutant/i-mutant2.0.html

6
https://zhanggroup.org/SSIPe/

7
https://www.rcsb.org/structure/1R42

8
http://bioinfo3d.cs.tau.ac.il/PatchDock/patchdock.html
