# Supplementary material for: Hotspot Mutations in SARS-CoV-2
Source: Front Genet. 2021 Nov 29;12:753440. doi: 10.3389/fgene.2021.753440 (PMC8667557; doi:10.3389/fgene.2021.753440)
Supplement: Supplementary file 1 [file DataSheet1.pdf]

# Supplementary to "Hotspot Mutations in SARS-CoV-2"

Indrajit Saha[a,d,*], Nimisha Ghosh[b,d], Nikhil Sharma[c], Suman Nandi[a]

[a] *Department of Computer Science and Engineering, National Institute of Technical Teachers' Training and Research, Kolkata, West Bengal, India*
[b] *Department of Computer Science and Information Technology, Institute of Technical Education and Research, Siksha 'O' Anusandhan (Deemed to be University), Bhubaneswar, Odisha, India*
[c] *Department of Electronics and Communication Engineering, Jaypee Institute of Information Technology, Noida, Uttar Pradesh, India*
[d] *Equally contributed*

Table S1: Statistics of sequences for different countries

| Name of the Country | Number of Sequences | Name of the Country | Number of Sequences | Name of the Country | Number of Sequences | Name of the Country | Number of Sequences | Name of the Country | Number of Sequences |
|---|---|---|---|---|---|---|---|---|---|
| USA | 11936 | China | 654 | Ecuador | 151 | Colombia | 53 | Georgia | 12 |
| England | 11687 | Portugal | 637 | Latvia | 149 | Romania | 50 | Mali | 11 |
| India | 10286 | Finland | 565 | Estonia | 147 | North Macedonia | 50 | Morocco | 11 |
| Scotland | 3784 | Luxembourg | 513 | Czech Republic | 141 | Sri Lanka | 44 | Kenya | 10 |
| Australia | 3345 | Canada | 496 | Aruba | 136 | Argentina | 41 | Malta | 10 |
| Denmark | 2544 | Ireland | 487 | Uganda | 130 | Senegal | 35 | Bosnia and Herzegovina | 4 |
| Wales | 2425 | Singapore | 477 | Egypt | 123 | Vietnam | 35 | Lebanon | 4 |
| Iceland | 1886 | Austria | 466 | Chile | 108 | Cambodia | 33 | Bulgaria | 4 |
| Belgium | 1534 | Northern Ireland | 426 | Nigeria | 94 | Tunisia | 31 | Cyprus | 4 |
| Switzerland | 1474 | Russia | 404 | Turkey | 93 | Costa Rica | 30 | Guatemala | 3 |
| Germany | 1366 | Indonesia | 314 | Peru | 90 | Kazakhstan | 29 | Kosovo | 3 |
| Spain | 1288 | Bangladesh | 296 | Slovenia | 90 | Kuwait | 27 | Iran | 3 |
| France | 1150 | Israel | 292 | Ghana | 82 | Montenegro | 25 | Jamaica | 3 |
| Brazil | 1072 | Mexico | 263 | Slovakia | 79 | Bahrain | 23 | Sierra Leone | 3 |
| Italy | 1034 | Jordan | 253 | Malaysia | 79 | Curacao | 22 | Rwanda | 2 |
| South Korea | 978 | Norway | 225 | Thailand | 69 | Pakistan | 19 | Brunei | 2 |
| Netherlands | 905 | Poland | 208 | Lithuania | 66 | Hungary | 17 | Panama | 1 |
| Japan | 738 | New Zealand | 205 | Croatia | 62 | Serbia | 16 | Nepal | 1 |
| South Africa | 715 | United Arab Emirates | 185 | Saudi Arabia | 61 | Belarus | 15 | | |
| Sweden | 665 | Greece | 151 | Oman | 59 | Suriname | 14 | | |

| Sl. No. | Name | Link |
|---|---|---|
| 1 | Monthwise Entropy of Mutations for 71038 Global SARS-CoV-2 Genomes | http://www.nitttrkol.ac.in/indrajit/projects/COVID-Hotspot-Mutation-Global-71K/downloads/supplementary/MonthWise-71038-Global-SARS-CoV-2-Mutation-Entropy.xlsx |
| 2 | Monthwise Entropy of Mutations for 10286 Indian SARS-CoV-2 Genomes | http://www.nitttrkol.ac.in/indrajit/projects/COVID-Hotspot-Mutation-Global-71K/downloads/supplementary/MonthWise-10286-Indian-SARS-CoV-2-Mutation-Entropy.xlsx |

Table S2: Link of Monthwise Entropy of 71038 Global and 10286 Indian SARS-CoV-2 Genomes

*Corresponding author: indrajit@nitttrkol.ac.in

# Table S3: Monthwise entropy of Hotspot mutations for 71038 Global SARS-CoV-2 Genomes along with other details

| Genomic Position | Change in Nucleotide | Change in Amino Acid | Gene | Month | January-February 2020 | March 2020 | April 2020 | May 2020 | June 2020 | July 2020 | August 2020 | September 2020 | October 2020 | November 2020 | December 2020 | January 2021 | February 2021 | March 2021 | April 2021 | May 2021 | June 2021 |
|---|---|---|---|---|---|---|---|---|---|---|---|---|---|---|---|---|---|---|---|---|---|
| | | | | Sequences | 1081 | 3820 | 3871 | 4291 | 4130 | 4167 | 4190 | 4173 | 3968 | 4017 | 4284 | 3853 | 4336 | 4892 | 5177 | 4044 | 6744 |
| 28881 | G>A, G>T | R203K/M | Nucleocapsid | | 0.17573 | 0.52284 | 0.57578 | 0.66947 | 0.71404 | 0.66292 | 0.69011 | 0.67615 | 0.56128 | 0.52351 | 0.54336 | 0.69275 | 0.84709 | 0.7677 | 0.68764 | 0.69886 | 0.93104 |
| 28883 | G>C | G204R | Nucleocapsid | | 0.17573 | 0.5126 | 0.57524 | 0.66934 | 0.71274 | 0.66621 | 0.69259 | 0.66658 | 0.55241 | 0.52056 | 0.53017 | 0.67157 | 0.68901 | 0.58783 | 0.60731 | 0.5686 | 0.63888 |
| 28882 | G>A | R203R | Nucleocapsid | | 0.17573 | 0.51124 | 0.57228 | 0.67097 | 0.69471 | 0.66054 | 0.69053 | 0.66816 | 0.55241 | 0.52089 | 0.53047 | 0.67345 | 0.69452 | 0.60284 | 0.60828 | 0.56638 | 0.64064 |
| 23604 | C>A, C>G | P681H/R | Spike | | 0.00737 | 0 | 0.01322 | 0.01058 | 0.03122 | 0.01309 | 0.02375 | 0.1695 | 0.14248 | 0.1709 | 0.38529 | 0.65383 | 0.86136 | 0.79124 | 0.68921 | 0.72314 | 0.96174 |
| 11296 | T>- | F108- | NSP6 | | 0 | 0 | 0.00442 | 0.00218 | 0 | 0.00224 | 0 | 0.00224 | 0.00853 | 0.03653 | 0.23789 | 0.57286 | 0.74539 | 0.72683 | 0.71361 | 0.53886 | 0.7748 |
| 21993 | A>- | Y144- | Spike | | 0.01908 | 0.01817 | 0.01483 | 0.01923 | 0.02265 | 0.01086 | 0.00413 | 0.01084 | 0.01366 | 0.0566 | 0.22766 | 0.52916 | 0.70969 | 0.62691 | 0.60525 | 0.75121 | 0.59473 |
| 11291 | G>- | G107- | NSP6 | | 0 | 0 | 0 | 0 | 0.00597 | 0.00224 | 0.01145 | 0.01614 | 0.00619 | 0.03653 | 0.24342 | 0.58116 | 0.72211 | 0.65088 | 0.67857 | 0.53067 | 0.77221 |
| 28280 | G>C | D3H | Nucleocapsid | | 0 | 0.00242 | 0.00239 | 0 | 0.00418 | 0.00415 | 0.00223 | 0.01239 | 0.01528 | 0.05982 | 0.22751 | 0.59907 | 0.83837 | 0.73197 | 0.71909 | 0.6122 | 0.49745 |
| 23063 | A>T | N501Y | Spike | | 0 | 0.00242 | 0 | 0.00622 | 0.0125 | 0.01985 | 0.0108 | 0.00592 | 0.04985 | 0.06347 | 0.24815 | 0.52987 | 0.69186 | 0.5938 | 0.60602 | 0.56821 | 0.6433 |
| 21770 | G>- | V70- | Spike | | 0.00737 | 0.00242 | 0.00442 | 0.00218 | 0.00644 | 0.01807 | 0.0909 | 0.08099 | 0.06453 | 0.16381 | 0.27068 | 0.58504 | 0.70488 | 0.66156 | 0.61217 | 0.58997 | 0.51484 |
| 3267 | C>T | T183I | NSP3 | | 0 | 0 | 0.00239 | 0.00404 | 0.017 | 0.05744 | 0.08683 | 0.06041 | 0.06439 | 0.11464 | 0.29502 | 0.52424 | 0.69331 | 0.60416 | 0.60303 | 0.58849 | 0.48222 |
| 11288 | T>- | S106- | NSP6 | | 0 | 0 | 0 | 0 | 0 | 0.00224 | 0 | 0.0139 | 0.00619 | 0.03653 | 0.23387 | 0.5356 | 0.69129 | 0.58399 | 0.60221 | 0.52781 | 0.66713 |
| 11289 | C>- | S106- | NSP6 | | 0 | 0 | 0 | 0.00404 | 0 | 0.00638 | 0.00413 | 0.0139 | 0.00619 | 0.03885 | 0.23667 | 0.54933 | 0.69366 | 0.59076 | 0.61173 | 0.52781 | 0.66879 |
| 21765 | T>- | I68- | Spike | | 0.01347 | 0.00242 | 0.00442 | 0 | 0.00226 | 0.00415 | 0.076 | 0.07115 | 0.07007 | 0.16522 | 0.27171 | 0.56158 | 0.69772 | 0.62899 | 0.6127 | 0.628 | 0.50829 |
| 21767 | C>- | H69- | Spike | | 0.01475 | 0.00448 | 0.00442 | 0.00218 | 0.00644 | 0.01231 | 0.076 | 0.07911 | 0.08581 | 0.17279 | 0.27185 | 0.56584 | 0.69248 | 0.61577 | 0.60727 | 0.58389 | 0.50546 |
| 11290 | T>- | S106- | NSP6 | | 0 | 0 | 0 | 0 | 0.00226 | 0.00224 | 0 | 0.0139 | 0.00619 | 0.03653 | 0.23604 | 0.5249 | 0.69167 | 0.59496 | 0.61431 | 0.52874 | 0.65628 |
| 21766 | A>- | I68- | Spike | | 0.00737 | 0 | 0.00442 | 0 | 0.00226 | 0.00415 | 0.076 | 0.07115 | 0.08279 | 0.16416 | 0.26829 | 0.56212 | 0.69248 | 0.61577 | 0.60744 | 0.58389 | 0.50566 |
| 21768 | A>- | H69- | Spike | | 0.00737 | 0 | 0.00442 | 0 | 0.00226 | 0.00415 | 0.076 | 0.07115 | 0.08045 | 0.16416 | 0.26829 | 0.56155 | 0.69252 | 0.61577 | 0.60727 | 0.58414 | 0.50404 |
| 21769 | T>- | H69- | Spike | | 0.01475 | 0 | 0.00682 | 0 | 0.00226 | 0.00415 | 0.076 | 0.07115 | 0.06217 | 0.15772 | 0.26711 | 0.55896 | 0.69253 | 0.62199 | 0.60744 | 0.58414 | 0.50566 |
| 11293 | T>- | G107- | NSP6 | | 0 | 0 | 0 | 0.00743 | 0 | 0.00224 | 0 | 0.00224 | 0.00619 | 0.03653 | 0.23387 | 0.51885 | 0.6939 | 0.60569 | 0.62028 | 0.52874 | 0.65294 |
| 11292 | G>- | G107- | NSP6 | | 0 | 0 | 0 | 0 | 0 | 0.00224 | 0 | 0.00224 | 0.00619 | 0.03653 | 0.23387 | 0.51651 | 0.69191 | 0.60008 | 0.61716 | 0.52874 | 0.65294 |
| 11294 | T>- | F108- | NSP6 | | 0 | 0 | 0 | 0 | 0 | 0.00224 | 0 | 0.00224 | 0.00619 | 0.03653 | 0.23387 | 0.51651 | 0.69191 | 0.60026 | 0.61747 | 0.52874 | 0.65294 |
| 24914 | G>C | D1118H | Spike | | 0 | 0 | 0 | 0.00404 | 0 | 0 | 0.00413 | 0 | 0.01452 | 0.03757 | 0.21467 | 0.53035 | 0.69677 | 0.61803 | 0.61307 | 0.59163 | 0.49764 |
| 6954 | T>C | I1412T | NSP3 | | 0.01908 | 0.00999 | 0 | 0.00404 | 0.00418 | 0.00593 | 0 | 0.00224 | 0.04197 | 0.04137 | 0.22319 | 0.51263 | 0.69835 | 0.61442 | 0.60626 | 0.59636 | 0.47689 |
| 28977 | C>T | S235F | Nucleocapsid | | 0 | 0 | 0.01052 | 0.00218 | 0.03319 | 0.06178 | 0.0413 | 0.06546 | 0.01366 | 0.05111 | 0.21784 | 0.51377 | 0.69308 | 0.61514 | 0.6091 | 0.59023 | 0.47911 |
| 21992 | T>- | Y144- | Spike | | 0.01908 | 0.01817 | 0.01949 | 0.02141 | 0.02265 | 0.00927 | 0.00413 | 0.01084 | 0.01528 | 0.05579 | 0.22235 | 0.51956 | 0.69515 | 0.62054 | 0.60593 | 0.60623 | 0.48684 |
| 913 | C>T | S36S | NSP2 | | 0 | 0 | 0.00239 | 0.00404 | 0.00226 | 0.00224 | 0 | 0.00985 | 0 | 0.0378 | 0.21334 | 0.53862 | 0.70657 | 0.62198 | 0.60635 | 0.59235 | 0.48429 |
| 11295 | T>- | F108- | NSP6 | | 0 | 0 | 0 | 0 | 0 | 0.00224 | 0 | 0.00224 | 0.00619 | 0.03653 | 0.23387 | 0.51651 | 0.69197 | 0.6134 | 0.62026 | 0.52874 | 0.65294 |
| 5986 | C>T | F1089F | NSP3 | | 0 | 0.00484 | 0.00682 | 0.00743 | 0.00226 | 0.00763 | 0.00759 | 0.02647 | 0.00796 | 0.07966 | 0.22879 | 0.51308 | 0.69832 | 0.61254 | 0.60457 | 0.58777 | 0.48267 |
| 28282 | T>A | D3E | Nucleocapsid | | 0 | 0 | 0 | 0 | 0 | 0.00224 | 0 | 0 | 0 | 0.03525 | 0.21117 | 0.54004 | 0.70525 | 0.6412 | 0.6304 | 0.58951 | 0.48382 |
| 28048 | G>T | R52I | ORF8 | | 0 | 0 | 0.00682 | 0.00808 | 0.01531 | 0.00987 | 0.00635 | 0.00638 | 0.01452 | 0.03906 | 0.21382 | 0.51334 | 0.69309 | 0.61632 | 0.60474 | 0.59197 | 0.47899 |
| 14676 | C>T | P412P | RdRp | | 0.01347 | 0 | 0.00442 | 0.0121 | 0.02491 | 0.00763 | 0.00413 | 0.00592 | 0.01294 | 0.06365 | 0.21581 | 0.51031 | 0.69508 | 0.61304 | 0.60618 | 0.58999 | 0.48267 |
| 23271 | C>A | A570D | Spike | | 0.01908 | 0.00242 | 0.00478 | 0 | 0.00226 | 0 | 0 | 0 | 0.00619 | 0.04222 | 0.21183 | 0.5136 | 0.70461 | 0.61152 | 0.60491 | 0.59023 | 0.47757 |
| 28281 | A>T | D3V | Nucleocapsid | | 0 | 0 | 0 | 0 | 0 | 0 | 0 | 0 | 0 | 0.03525 | 0.21117 | 0.543 | 0.71087 | 0.64204 | 0.63569 | 0.59314 | 0.48217 |
| 27972 | C>T | Q27* | ORF8 | | 0 | 0.00242 | 0.00239 | 0.00404 | 0.0207 | 0.0121 | 0.01303 | 0.00224 | 0.00667 | 0.04386 | 0.2175 | 0.51576 | 0.69508 | 0.61411 | 0.60474 | 0.58777 | 0.47757 |
| 5388 | C>A | A890D | NSP3 | | 0 | 0 | 0 | 0 | 0 | 0 | 0 | 0.00224 | 0.02142 | 0.03397 | 0.21334 | 0.51254 | 0.6931 | 0.61339 | 0.60652 | 0.59023 | 0.47943 |
| 28111 | A>G | Y73C | ORF8 | | 0 | 0 | 0.00239 | 0.00218 | 0.00769 | 0 | 0 | 0.00224 | 0 | 0.03525 | 0.2105 | 0.50821 | 0.69511 | 0.61169 | 0.60652 | 0.58801 | 0.49014 |
| 23709 | C>T | T716I | Spike | | 0 | 0.00242 | 0.00442 | 0.00218 | 0.00226 | 0.00224 | 0 | 0.00224 | 0.00433 | 0.04031 | 0.21732 | 0.51239 | 0.6931 | 0.61148 | 0.60491 | 0.58608 | 0.48134 |
| 24506 | T>G | S982A | Spike | | 0 | 0 | 0 | 0 | 0 | 0 | 0 | 0 | 0 | 0.03397 | 0.21117 | 0.50751 | 0.69513 | 0.61304 | 0.60635 | 0.58801 | 0.48178 |
| 15279 | C>T | H613H | RdRp | | 0 | 0 | 0 | 0.00218 | 0 | 0 | 0.00413 | 0.00447 | 0.00234 | 0.03525 | 0.21652 | 0.50961 | 0.69838 | 0.61204 | 0.60491 | 0.59071 | 0.47801 |
| 16176 | T>C | T912T | RdRp | | 0 | 0.00242 | 0 | 0 | 0 | 0 | 0 | 0 | 0 | 0.03397 | 0.21117 | 0.50821 | 0.6931 | 0.61169 | 0.60491 | 0.59023 | 0.47801 |
| 21991 | T>- | V143- | Spike | | 0.01908 | 0.01817 | 0.01949 | 0.02059 | 0.02491 | 0.00927 | 0.00413 | 0.01239 | 0.01294 | 0.05464 | 0.22235 | 0.52189 | 0.69315 | 0.61989 | 0.6061 | 0.62763 | 0.42016 |
| 25563 | G>T | Q57H | ORF3a | | 0.11325 | 0.50377 | 0.60476 | 0.67212 | 0.68139 | 0.4948 | 0.4755 | 0.55943 | 0.51104 | 0.6088 | 0.61668 | 0.664 | 0.4925 | 0.27796 | 0.07501 | 0.16607 | 0.17795 |
| 22227 | C>T | A222V | Spike | | 0.00737 | 0.00448 | 0 | 0.00622 | 0.01159 | 0.00593 | 0.32834 | 0.55355 | 0.6912 | 0.64399 | 0.63737 | 0.37683 | 0.30797 | 0.08118 | 0.22823 | 0.15285 | 0.26379 |
| 28253 | C>T, C>- | F120F/- | ORF8 | | 0.05866 | 0.0283 | 0.04443 | 0.04346 | 0.01925 | 0.04592 | 0.06039 | 0.12842 | 0.10018 | 0.10511 | 0.13092 | 0.17164 | 0.17927 | 0.18013 | 0.67424 | 0.64139 | 0.89562 |

# Table S4: Monthwise entropy of Hotspot mutations for 10286 Indian SARS-CoV-2 Genomes along with other details

| Genomic Position | Change in Nucleotide | Change in Amino Acid | Gene | Month | January-March 2020 | April 2020 | May 2020 | June 2020 | July 2020 | August 2020 | September 2020 | October 2020 | November 2020 | December 2020 | January 2021 | February 2021 | March 2021 | April 2021 | May 2021 | June 2021 |
|---|---|---|---|---|---|---|---|---|---|---|---|---|---|---|---|---|---|---|---|---|
| | | | | Sequences | 191 | 433 | 958 | 975 | 678 | 625 | 610 | 351 | 296 | 620 | 298 | 731 | 1259 | 1530 | 309 | 422 |
| 28881 | G>A, G>T | R203K/M | Nucleocapsid | | 0.38864 | 0.52778 | 0.66332 | 0.72084 | 0.86692 | 0.79288 | 0.71412 | 0.73609 | 0.69966 | 0.72497 | 0.85964 | 1.13735 | 1.03419 | 0.49447 | 0.51329 | 0.13596 |
| 23604 | C>A, C>G | P681H/R | Spike | | 0 | 0 | 0 | 0.00807 | 0.06157 | 0.04647 | 0.22658 | 0.15811 | 0.20879 | 0.48517 | 0.67263 | 1.00026 | 1.00757 | 0.45975 | 0.2105 | 0.0954 |
| 28882 | G>A | R203R | Nucleocapsid | | 0.38864 | 0.52778 | 0.66332 | 0.70671 | 0.85701 | 0.78243 | 0.68816 | 0.69266 | 0.70076 | 0.6816 | 0.74858 | 0.68423 | 0.72919 | 0.35878 | 0.09527 | 0.05869 |
| 28883 | G>C | G204R | Nucleocapsid | | 0.38864 | 0.52778 | 0.66332 | 0.71998 | 0.85696 | 0.7911 | 0.67807 | 0.69266 | 0.69966 | 0.68091 | 0.72857 | 0.66549 | 0.72686 | 0.35546 | 0.09527 | 0.04208 |
| 26767 | T>C, T>G | I82T/S | Membrane | | 0 | 0 | 0 | 0 | 0 | 0 | 0 | 0 | 0 | 0.02167 | 0.07831 | 0.50086 | 0.87744 | 0.87712 | 0.3797 | 0.12609 |
| 28253 | C>T, C>- | F120F/- | ORF8 | | 0 | 0.09204 | 0.04813 | 0.01472 | 0.04713 | 0.07717 | 0.13269 | 0.10823 | 0.15801 | 0.24618 | 0.26037 | 0.31556 | 0.40588 | 1.24563 | 1.36599 | 0.61455 |
| 25469 | C>T | S26L | ORF3a | | 0 | 0 | 0 | 0 | 0 | 0 | 0 | 0 | 0 | 0.03886 | 0.12296 | 0.67835 | 0.77148 | 0.43614 | 0.19327 | 0.08412 |
| 29402 | G>T | D377Y | Nucleocapsid | | 0 | 0.01626 | 0.04337 | 0.02279 | 0.02008 | 0.03861 | 0.08343 | 0.06203 | 0.13544 | 0.16856 | 0.21076 | 0.66242 | 0.71068 | 0.40394 | 0.21478 | 0.08412 |
| 22917 | T>G | L452R | Spike | | 0 | 0 | 0 | 0 | 0 | 0 | 0.01212 | 0.01945 | 0 | 0.06937 | 0.17942 | 0.61553 | 0.69128 | 0.41187 | 0.17363 | 0.08412 |
| 27638 | T>C | V82A | ORF7a | | 0.03244 | 0 | 0.00819 | 0 | 0.01106 | 0 | 0 | 0 | 0 | 0.03886 | 0.12296 | 0.61853 | 0.70268 | 0.40396 | 0.26518 | 0.12348 |
| 25563 | G>T | Q57H | ORF3a | | 0.24675 | 0.39695 | 0.63645 | 0.65383 | 0.69241 | 0.62203 | 0.60196 | 0.6352 | 0.69312 | 0.70034 | 0.69089 | 0.61853 | 0.27758 | 0.07785 | 0.06051 | 0.01662 |
| 22444 | C>T | D294D | Spike | | 0.18848 | 0.26854 | 0.45432 | 0.64589 | 0.60158 | 0.62823 | 0.57137 | 0.63652 | 0.71301 | 0.72078 | 0.66875 | 0.60384 | 0.33564 | 0.14461 | 0.076 | 0 |
| 18877 | C>T | L280L | Exon | | 0.21862 | 0.38835 | 0.54722 | 0.64359 | 0.66329 | 0.60937 | 0.56412 | 0.63717 | 0.69279 | 0.68991 | 0.69229 | 0.59521 | 0.26478 | 0.0722 | 0.02167 | 0.01662 |
| 26735 | C>T | Y71Y | Membrane | | 0.18848 | 0.38398 | 0.56048 | 0.64023 | 0.65438 | 0.61341 | 0.55872 | 0.63717 | 0.69294 | 0.68991 | 0.66875 | 0.59264 | 0.26153 | 0.06961 | 0.02167 | 0.01662 |
| 28854 | C>T | S194L | Nucleocapsid | | 0.22066 | 0.27425 | 0.44973 | 0.6125 | 0.61506 | 0.61734 | 0.56766 | 0.63115 | 0.71324 | 0.68937 | 0.67686 | 0.59521 | 0.26963 | 0.06047 | 0.04332 | 0.01662 |
| 24410 | G>A | D950N | Spike | | 0 | 0 | 0 | 0 | 0 | 0 | 0 | 0 | 0 | 0 | 0.02234 | 0.13658 | 0.39442 | 0.77514 | 0.8066 | 0.44966 |
| 21987 | G>A | G142D | Spike | | 0 | 0 | 0 | 0.02083 | 0 | 0 | 0 | 0 | 0 | 0.03054 | 0.04467 | 0.66005 | 0.82831 | 0.81174 | 0.45129 | 0.86405 |
| 21618 | C>G | T19R | Spike | | 0 | 0 | 0 | 0.00807 | 0 | 0 | 0 | 0 | 0 | 0.03054 | 0.02234 | 0.13931 | 0.40908 | 0.6633 | 0.32397 | 0.12879 |
| 27752 | C>T | T120I | ORF7a | | 0 | 0 | 0.02314 | 0.0289 | 0 | 0.01187 | 0.02197 | 0 | 0.04027 | 0.02389 | 0.04005 | 0.03388 | 0.36483 | 0.66234 | 0.42698 | 0.12879 |
| 22034 | A>- | R158- | Spike | | 0 | 0 | 0 | 0 | 0 | 0 | 0 | 0 | 0 | 0.02167 | 0.02234 | 0.13002 | 0.31882 | 0.91167 | 0.95863 | 0.88102 |
| 22995 | C>A | T478K | Spike | | 0 | 0 | 0 | 0.00807 | 0 | 0.01187 | 0 | 0.03496 | 0.02247 | 0 | 0.02234 | 0.03388 | 0.368 | 0.67377 | 0.36956 | 0.12879 |
| 28461 | A>G | D63G | Nucleocapsid | | 0 | 0 | 0 | 0 | 0 | 0 | 0 | 0 | 0 | 0 | 0.02234 | 0.03695 | 0.34688 | 0.6977 | 0.83147 | 0.12879 |
| 15451 | G>A | G671S | RdRp | | 0 | 0.08241 | 0.07613 | 0 | 0 | 0.03033 | 0.05952 | 0.07436 | 0 | 0.03886 | 0.02234 | 0.04082 | 0.33623 | 0.71838 | 0.61542 | 0.3333 |
| 23012 | G>C | E484Q | Spike | | 0.03244 | 0 | 0.00819 | 0.02279 | 0.02008 | 0 | 0.03096 | 0.01945 | 0 | 0.06842 | 0.25523 | 0.86935 | 0.91586 | 0.60253 | 0.30151 | 0.10421 |
| 22033 | C>- | F157- | Spike | | 0 | 0 | 0 | 0 | 0 | 0.01187 | 0 | 0 | 0.02247 | 0.02167 | 0.06237 | 0.1355 | 0.307 | 0.81401 | 0.75382 | 0.43862 |
| 16466 | C>T | P77L | Helicase | | 0 | 0 | 0 | 0 | 0.01106 | 0.01187 | 0.01212 | 0 | 0 | 0 | 0.04005 | 0.03388 | 0.3135 | 0.69498 | 0.62061 | 0.3333 |
| 22032 | T>- | F157- | Spike | | 0 | 0 | 0 | 0 | 0 | 0 | 0 | 0 | 0 | 0.02167 | 0.02234 | 0.15165 | 0.31075 | 0.79675 | 0.68695 | 0.25559 |
| 11201 | A>G | T77A | NSP6 | | 0 | 0 | 0 | 0 | 0 | 0 | 0 | 0 | 0 | 0.03886 | 0.12296 | 0.59135 | 0.68439 | 0.66024 | 0.72325 | 0.67339 |
| 28249 | A>- | D119- | ORF8 | | 0 | 0 | 0.03518 | 0 | 0 | 0 | 0 | 0 | 0 | 0 | 0.02234 | 0.04541 | 0.21405 | 0.8722 | 0.87319 | 0.48772 |
| 5184 | C>T | P822L | NSP3 | | 0.03244 | 0 | 0.01494 | 0 | 0 | 0 | 0 | 0.01945 | 0.02247 | 0.03361 | 0 | 0.06773 | 0.35666 | 0.70071 | 0.70426 | 0.68023 |
| 22031 | T>- | F157- | Spike | | 0 | 0 | 0 | 0 | 0 | 0 | 0 | 0 | 0 | 0.02167 | 0.02234 | 0.12517 | 0.29422 | 0.6956 | 0.67249 | 0.32818 |
| 313 | C>T | L16L | NSP1 | | 0.12021 | 0.43352 | 0.54325 | 0.56205 | 0.66626 | 0.61073 | 0.60436 | 0.27011 | 0.29612 | 0.24295 | 0.1572 | 0.27327 | 0.18171 | 0.02414 | 0.02167 | 0.01662 |
| 22029 | A>- | E156- | Spike | | 0 | 0 | 0 | 0 | 0 | 0 | 0 | 0 | 0 | 0.03361 | 0 | 0.12517 | 0.28858 | 0.68872 | 0.71225 | 0.5267 |
| 5700 | C>A | A994D | NSP3 | | 0.03244 | 0.38398 | 0.51124 | 0.55287 | 0.66143 | 0.60522 | 0.59848 | 0.26765 | 0.28818 | 0.22109 | 0.12296 | 0.26647 | 0.18414 | 0.0323 | 0 | 0.02997 |
| 20396 | A>G | K259R | endoRNAse | | 0 | 0 | 0 | 0 | 0 | 0 | 0 | 0 | 0.02247 | 0.0508 | 0.11074 | 0.58873 | 0.68606 | 0.62059 | 0.58042 | 0.29616 |
| 3267 | C>T | T183I | NSP3 | | 0 | 0 | 0.00819 | 0.05235 | 0.23578 | 0.35323 | 0.25885 | 0.38157 | 0.56417 | 0.5948 | 0.48622 | 0.58921 | 0.61979 | 0.33473 | 0.08235 | 0.04208 |
| 22030 | G>- | E156- | Spike | | 0 | 0.01626 | 0 | 0 | 0 | 0 | 0 | 0 | 0 | 0.02167 | 0.02234 | 0.12517 | 0.29496 | 0.68183 | 0.71207 | 0.61935 |
| 28251 | T>- | F120- | ORF8 | | 0 | 0.01626 | 0.02115 | 0.00807 | 0.02833 | 0.01187 | 0.04741 | 0.01945 | 0.02247 | 0.02389 | 0.056 | 0.0473 | 0.22348 | 0.6788 | 0.70658 | 0.4843 |
| 28248 | G>- | D119- | ORF8 | | 0 | 0 | 0.02699 | 0 | 0 | 0 | 0 | 0 | 0 | 0 | 0.02234 | 0.02919 | 0.21817 | 0.70329 | 0.72188 | 0.48772 |
| 24775 | A>T | Q1071H | Spike | | 0 | 0 | 0 | 0 | 0 | 0 | 0 | 0 | 0 | 0.01195 | 0.09598 | 0.75205 | 0.79963 | 0.57275 | 0.22214 | 0.02997 |
| 21895 | T>C | D111D | Spike | | 0 | 0 | 0 | 0 | 0 | 0 | 0 | 0 | 0 | 0.03886 | 0.11074 | 0.58607 | 0.76731 | 0.57702 | 0.35523 | 0.15819 |
| 28280 | G>C | D3H | Nucleocapsid | | 0 | 0 | 0 | 0 | 0.01106 | 0.01187 | 0.0551 | 0.07436 | 0.12538 | 0.28909 | 0.37997 | 0.63764 | 0.87162 | 0.52641 | 0.18336 | 0.08411 |
| 28250 | T>- | D119- | ORF8 | | 0 | 0.01626 | 0.02699 | 0 | 0.01106 | 0.01187 | 0 | 0 | 0 | 0 | 0.02234 | 0.03766 | 0.20764 | 0.67033 | 0.68817 | 0.4843 |
| 28252 | T>- | F120- | ORF8 | | 0 | 0.01626 | 0.02115 | 0 | 0.02008 | 0.01187 | 0 | 0 | 0 | 0.01195 | 0.04005 | 0.02073 | 0.20535 | 0.66742 | 0.68526 | 0.48772 |
| 11418 | T>C | V149A | NSP6 | | 0 | 0 | 0 | 0 | 0 | 0 | 0 | 0.01945 | 0.02247 | 0.01195 | 0.02234 | 0.12026 | 0.29422 | 0.70978 | 0.67349 | 0.6931 |
| 9891 | C>T | A446V | NSP4 | | 0 | 0 | 0 | 0 | 0.01106 | 0 | 0.01212 | 0 | 0 | 0.04678 | 0.04467 | 0.02659 | 0.30618 | 0.69815 | 0.64774 | 0.69297 |
| 17523 | G>T | M429I | Helicase | | 0 | 0 | 0 | 0 | 0 | 0 | 0 | 0.01945 | 0.02247 | 0.04678 | 0.133 | 0.58873 | 0.67007 | 0.51775 | 0.23182 | 0.06408 |
| 3457 | C>T | Y246Y | NSP3 | | 0 | 0 | 0 | 0 | 0 | 0 | 0.02423 | 0.01945 | 0 | 0.03886 | 0.11074 | 0.58873 | 0.68037 | 0.51678 | 0.17363 | 0.0743 |
| 4965 | C>T | T749I | NSP3 | | 0 | 0 | 0 | 0 | 0 | 0.01187 | 0 | 0 | 0 | 0.03886 | 0.12296 | 0.59004 | 0.66477 | 0.52738 | 0.20275 | 0.06408 |
| 22022 | G>A | E154K | Spike | | 0 | 0 | 0 | 0 | 0 | 0 | 0 | 0 | 0 | 0.06051 | 0.09311 | 0.57455 | 0.78879 | 0.50022 | 0.0904 | 0.08411 |
| 1191 | C>T | P129L | NSP2 | | 0 | 0.01626 | 0.02115 | 0.00807 | 0.02833 | 0.01187 | 0.02423 | 0.04898 | 0.05631 | 0.09427 | 0.1784 | 0.10507 | 0.28477 | 0.64774 | 0.63273 | 0.65254 |
| 21846 | C>T | T95I | Spike | | 0 | 0 | 0 | 0 | 0.02008 | 0.02152 | 0.01212 | 0.01945 | 0.02247 | 0.08235 | 0.07081 | 0.44354 | 0.59462 | 0.46015 | 0.624 | 0.66984 |

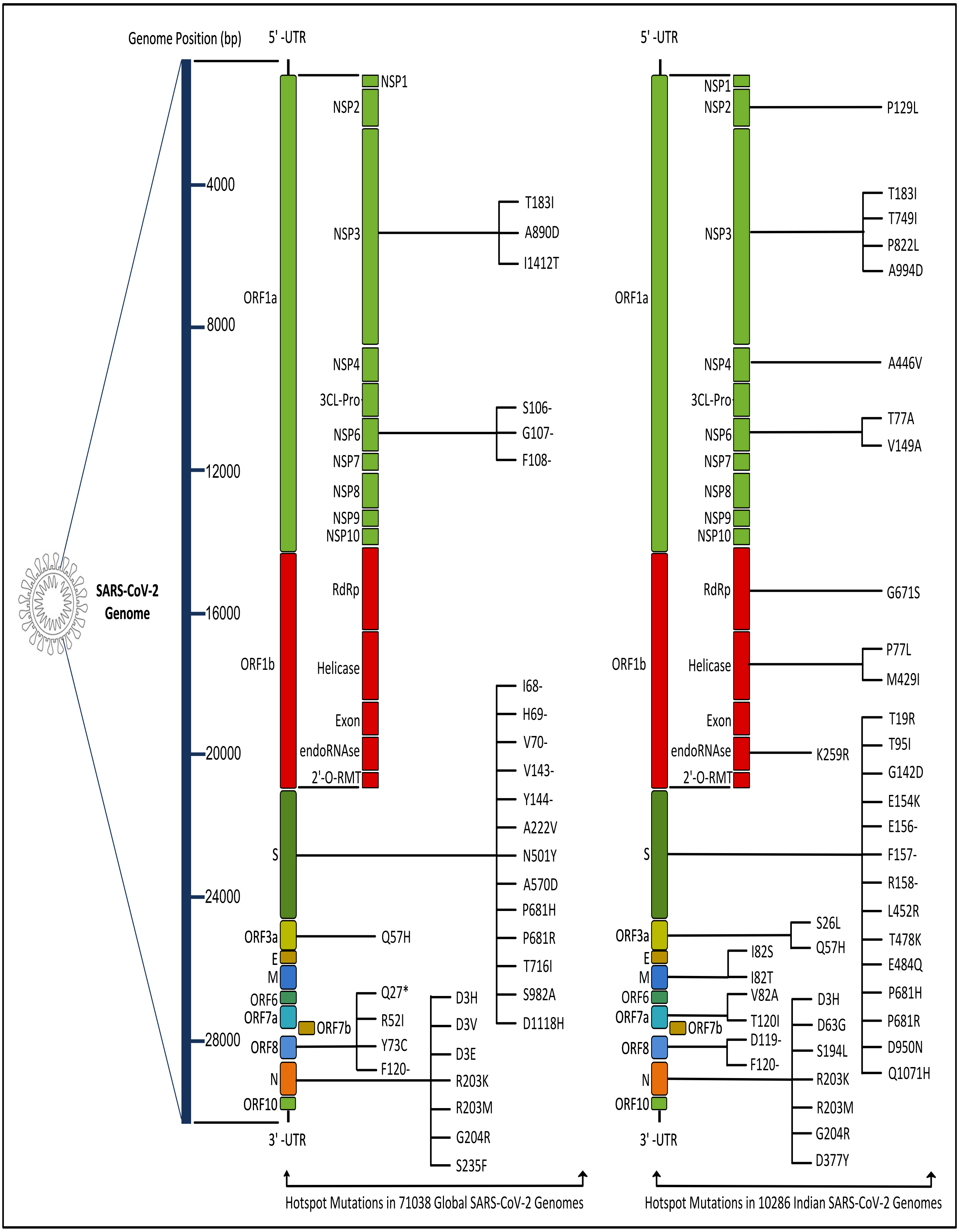


Figure S1: Amino acid changes in the proteins for the non-synonymous deletions and substitutions

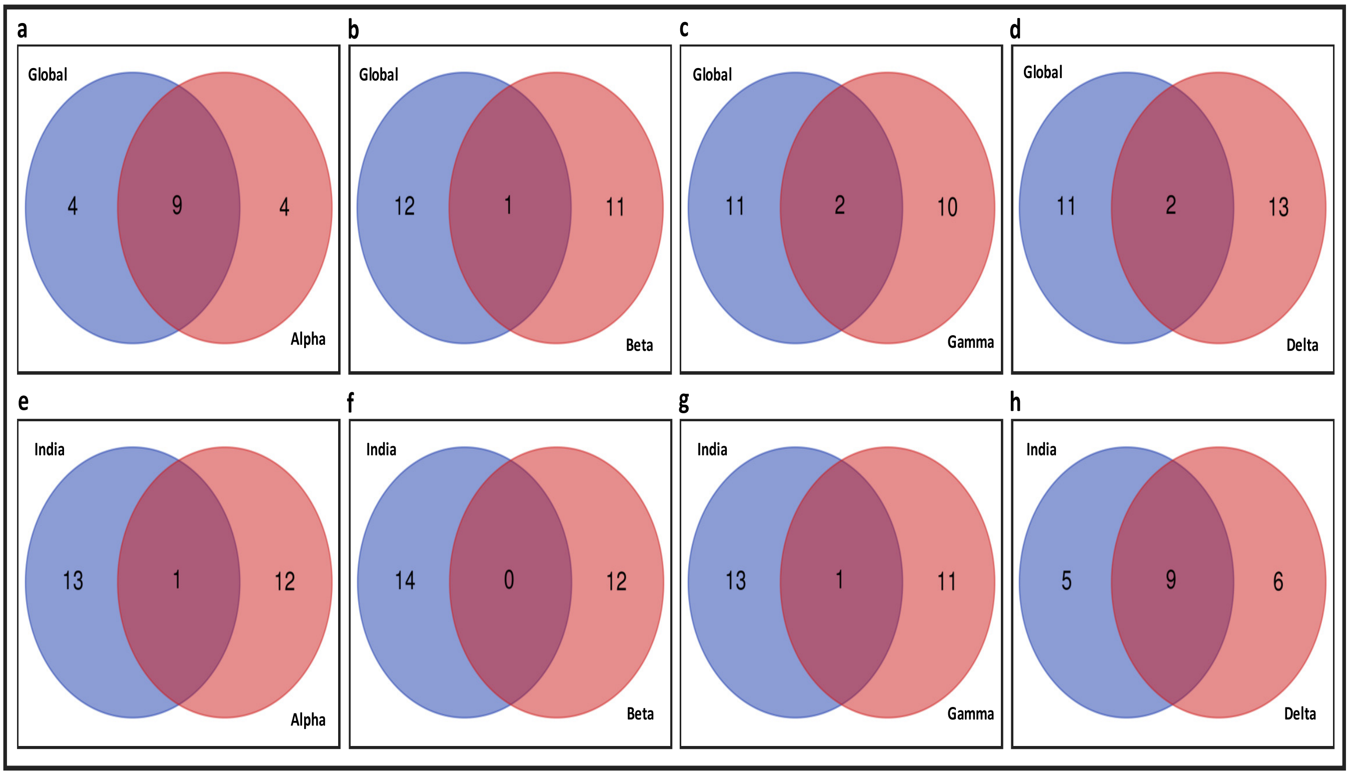


Figure S2: Venn diagrams to represent common hotspot mutations in the SARS-CoV-2 genomes for (a) Global vs. Alpha (b) Global vs. Beta (c) Global vs. Gamma (d) Global vs. Delta (e) India vs. Alpha (f) India vs. Beta (g) India vs. Gamma (h) India vs. Delta

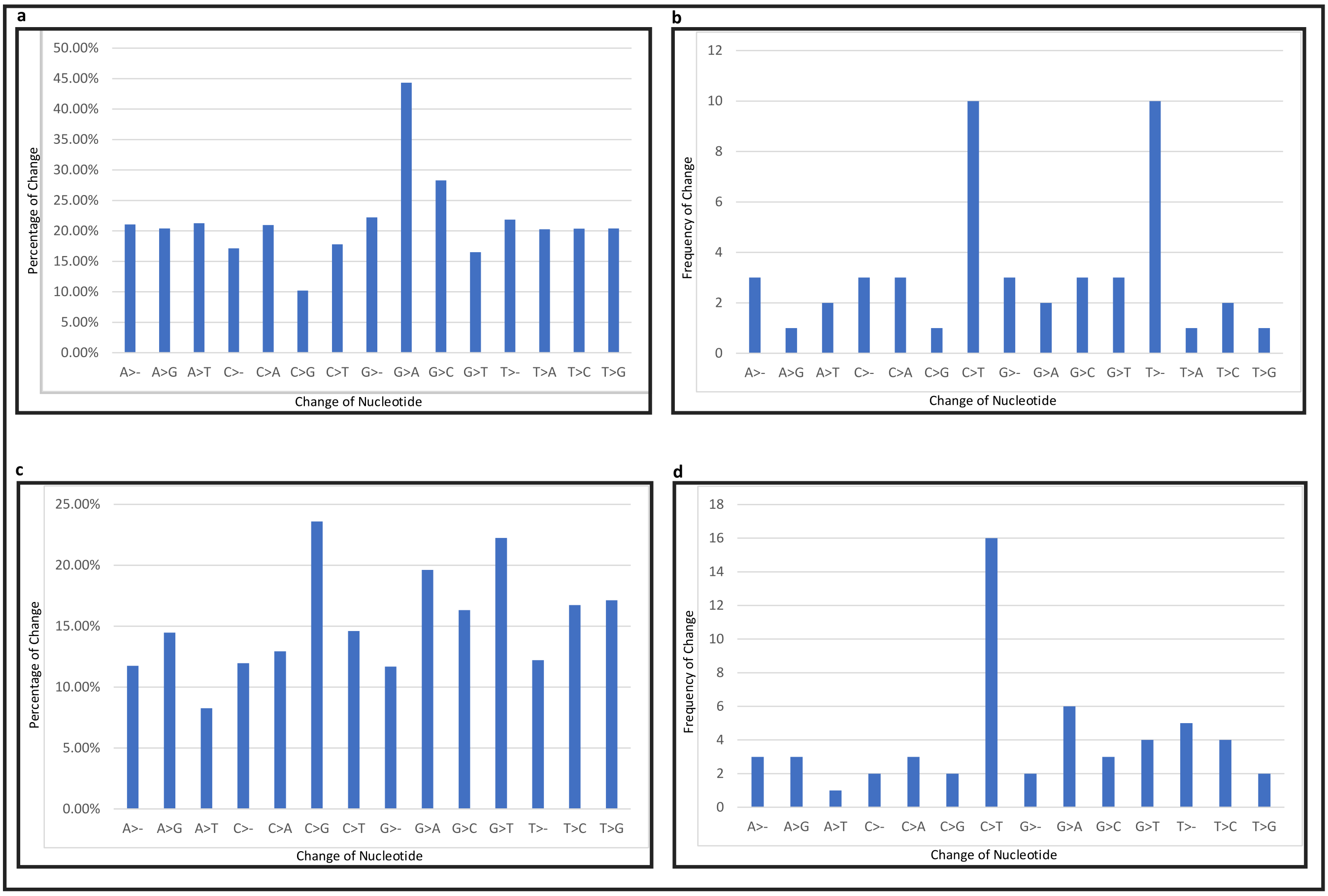


Figure S3: (a) Percentage of Nucleotide change (b) Frequency of Nucleotide change for hotspot mutations in Global SARS-CoV-2 genomes (c) Percentage of Nucleotide change and (d) Frequency of Nucleotide change for hotspot mutations in Indian SARS-CoV-2 genomes
